# The cnidarian-bilaterian ancestor possessed at least 56 homeoboxes: evidence from the starlet sea anemone, *Nematostella vectensis*

**DOI:** 10.1186/gb-2006-7-7-r64

**Published:** 2006-07-24

**Authors:** Joseph F Ryan, Patrick M Burton, Maureen E Mazza, Grace K Kwong, James C Mullikin, John R Finnerty

**Affiliations:** 1 Bioinformatics Program, Boston University, Cummington Street, Boston, MA 02215, USA; 2 National Human Genome Research Institute, Fishers Lane, Bethesda, MD 20892, USA; 3 Department of Biology, Boston University, Cummington Street, Boston, MA 02215, USA

## Abstract

**Background:**

Homeodomain transcription factors are key components in the developmental toolkits of animals. While this gene superclass predates the evolutionary split between animals, plants, and fungi, many homeobox genes appear unique to animals. The origin of particular homeobox genes may, therefore, be associated with the evolution of particular animal traits. Here we report the first near-complete set of homeodomains from a basal (diploblastic) animal.

**Results:**

Phylogenetic analyses were performed on 130 homeodomains from the sequenced genome of the sea anemone *Nematostella vectensis *along with 228 homeodomains from human and 97 homeodomains from *Drosophila*. The *Nematostella *homeodomains appear to be distributed among established homeodomain classes in the following fashion: 72 ANTP class; one HNF class; four LIM class; five POU class; 33 PRD class; five SINE class; and six TALE class. For four of the *Nematostella *homeodomains, there is disagreement between neighbor-joining and Bayesian trees regarding their class membership. A putative *Nematostella *CUT class gene is also identified.

**Conclusion:**

The homeodomain superclass underwent extensive radiations prior to the evolutionary split between Cnidaria and Bilateria. Fifty-six homeodomain families found in human and/or fruit fly are also found in *Nematostella*, though seventeen families shared by human and fly appear absent in *Nematostella*. Homeodomain loss is also apparent in the bilaterian taxa: eight homeodomain families shared by *Drosophila *and *Nematostella *appear absent from human (CG13424, EMXLX, HOMEOBRAIN, MSXLX, NK7, REPO, ROUGH, and UNC4), and six homeodomain families shared by human and *Nematostella *appear absent from fruit fly (ALX, DMBX, DUX, HNF, POU1, and VAX).

## Abstract 2

The first near-complete set of homeodomains from a non-bilaterian animal is described.

## Background

Homeobox genes constitute an ancient superclass of regulatory genes with diverse developmental functions [1]. The homeobox, which encodes a helix-turn-helix DNA-binding motif known as the homeodomain, originated prior to the evolutionary split between plants, fungi, and metazoans [2]. The homeodomain is commonly 60 amino acids in length, though recognizable homeodomains may be as long as 97 or as short as 54 amino acids (reviewed in [3]).

Based on phylogenetic analyses and chromosomal mapping studies, animal homeodomains can be divided among ten distinct classes: ANTP, CUT, HNF, LIM, POU, PRD, PROS, SINE, TALE, and ZF [3-16]. The ANTP and PRD classes are substantially larger than the other classes, and these two classes are thought to be sister clades [5,7]. Within the ANTP class, there is evidence for a monophyletic subclass comprising Hox-related genes [4,7]. The PRD class can be divided into subclasses based on the amino acid present at position 50 of the homeodomain (Q50, K50, or S50), but these subclasses do no not appear to represent monophyletic groups [5,7]. The remaining eight homeodomain classes are significantly smaller than the ANTP and PRD classes, and they are thought to have emerged as a series of lineages basal to an ANTP-PRD clade [6]. To this point, the HNF class has only been reported from vertebrates [6]. Structural and functional properties of the homeodomain appear largely conserved within these homeodomain classes [4]. The homeodomain sequences encoded by orthologous homeobox genes are often so highly conserved that orthology between protostomes and deuterostomes, and even between bilaterians and non-bilaterians, is readily apparent [17].

**Figure 1:**
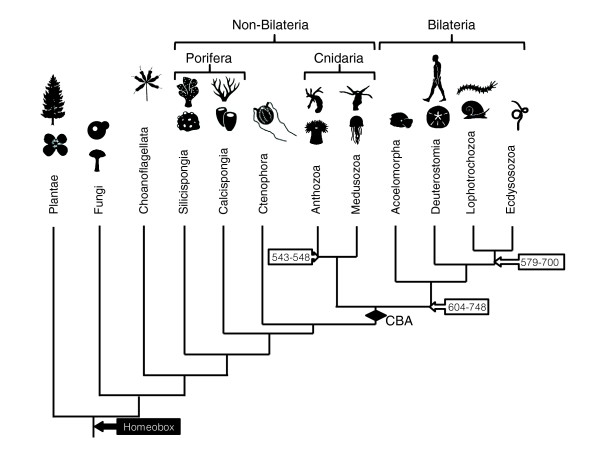
Phylogenetic relationships among major metazoan lineages. The topology of the tree is consistent with several recent molecular phylogenetic analyses [100-106]. Estimated divergence times for Cnidaria versus Bilateria, protostomes versus deuterostomes, and lophotrochozoans versus ecdysozoans are indicated in the white boxes [18]. The origin of the homeobox gene superclass must have predated the split between animals, plants, and fungi.

The ANTP, PRD, CUT, LIM, POU, PROS, SINE, TALE, and ZF classes are known from both protostome and deuterostome metazoans [3]. Therefore, we can trace their origins to the protostome-deuterostome ancestor, which a recent estimate places at some 579 to 700 million years ago (Figure 1) [18]. Identification of these homeobox classes in outgroup taxa would indicate even greater antiquity. For example, molecular clock estimates based on maximum likelihood and minimum evolution suggest that the cnidarian-bilaterian divergence predated the protostome-deuterostome divergence by 25 to 48 million years [18].

Establishing the antiquity of homeobox genes is critical to understanding the role of these genes in metazoan evolution. The functional diversification of homeobox genes, by gene duplication and divergence, or by cis-regulatory evolution, has been touted as an important mechanism in the evolution of diverse body plans and organs in bilaterian metazoans [6,19-25]. The Cnidaria is the likely sister group of the Bilateria [26,27], and since their divergence from a common ancestor, these two lineages have undergone very different evolutionary trajectories (Figure 1). The bilaterian ancestor has spawned over 30 distinct phyla comprising more than one million extant species; the cnidarian ancestor has spawned some 10,000 extant species, all comfortably housed in a single phylum [28]. The maximum complexity and morphological diversity of cnidarian body plans (for example, sea anemones, sea pens, corals, hydras, and jellyfishes) is modest when compared to the maximum complexity and morphological diversity of bilaterian body plans (for example, vertebrates, sea squirts, sea urchins, insects, nematodes, octopi, and phoronids [25,29]). Taking into account the presumed importance of homeobox genes in the morphological diversification of bilaterians, the close evolutionary relationship between the Bilateria and the Cnidaria, and the contrasting evolutionary trajectories of these two lineages, a comparison of cnidarians and bilaterians becomes critical for understanding the significance of homeobox genes in the morphological diversification of animal body plans.

Here, we seek to identify homeobox genes that were present in the cnidarian-bilaterian ancestor using phylogenetic analysis of homeodomains from bilaterians and cnidarians. Our analysis takes advantage of the curated genomic datasets of the fruit fly *Drosophila melanogaster *[30-34] and *Homo sapiens *[35,36] as well as the recently completed rough draft of the sea anemone *Nematostella vectensis*, a representative cnidarian (Joint Genome Institute; D Rokhsar, principal investigator).

**Figure 2:**
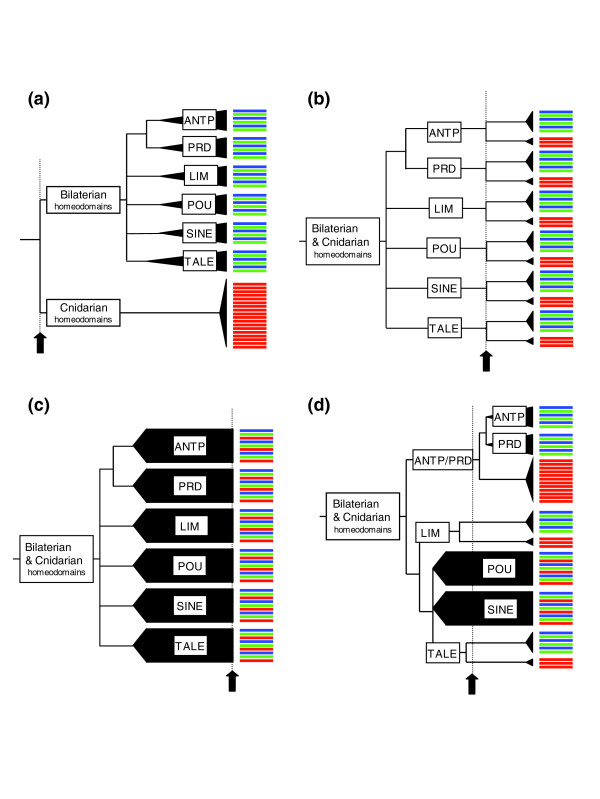
Hypothetical scenarios for the evolution and diversification of homeodomain classes relative to the cnidarian-bilaterian divergence. The timing of the cnidarian-bilaterian divergence is indicated by an arrow and a dashed vertical line. Cnidarian homeobox genes are indicated by red lines. Protostome (for example, *Drosophila*) homeobox genes are indicated by green lines. Deuterostome (for example, human) homeobox genes are indicated by blue lines. **(a) **Cnidaria diverges from Bilateria prior to origin of the major homeodomain classes (ANTP, PRD, LIM, POU, SINE, TALE). **(b) **Cnidaria diverges from Bilateria after the origin of homeodomain classes but before their diversification. **(c) **Cnidaria diverges from Bilateria after the diversification of homeobox classes. **(d) **At the time of the cnidarian-bilaterian divergence, some homeobox classes have not yet originated (ANTP, PRD) whereas others have diversified extensively (POU, SINE).

The phylogenetic analyses presented here reveal the extent to which the homeobox gene superclass had radiated prior to the evolutionary split between Cnidaria and Bilateria. For example, at one extreme, the Cnidaria could have diverged from the Bilateria prior to the origin of the aforementioned homeobox classes (ANTP, PRD, LIM, POU, and so on). If so, then the cnidarian homeobox genes and the bilaterian homeobox genes would constitute independent radiations on the phylogeny (Figure 2a). This possibility is ruled out by published studies that have identified distinct ANTP, POU, PRD, and SINE homeodomains in the Cnidaria [5,17,37-45]. Alternatively, the Cnidaria could have diverged from the Bilateria after the origin of the class founder genes (for example, the ancestral ANTP class gene, the ancestral PRD class gene, and so on), but prior to the subsequent radiations of these classes. In this case, the cnidarian and bilaterian class radiations would constitute mutually exclusive monophyletic groups (Figure 2b). However, if the homeobox classes had undergone extensive radiations prior to the cnidarian-bilaterian divergence, then the same homeobox families would be represented in cnidarian and bilaterian genomes (Figure 2c). Finally, it might also be the case that some homeobox classes had radiated prior to the cnidarian-bilaterian radiation, while other classes had not (Figure 2d).

The phylogenetic analyses presented here reveal that the ANTP, PRD, LIM, SINE, and POU classes had radiated extensively prior to the divergence of the Cnidaria and the Bilateria. The HNF class, formerly known only from vertebrates, is also represented in the *Nematostella *genome. In addition, we identify a putative CUT class gene in *Nematostella *by searching the predicted gene database at StellaBase [46,47]. Our analyses fail to identify ZF or PROS homeodomains in *Nematostella*. The phylogenetic analyses reveal 56 distinct homeodomain families that appear to be shared by *Nematostella *and one or both of the bilaterian taxa.

## Results

### Metazoan homeodomains

We retrieved 455 distinct homeodomains from the three metazoan taxa under study, including 130 from the genome of *Nematostella*, a representative non-bilaterian, 228 from *Homo*, a representative deuterostome bilaterian, and 97 from *Drosophila*, a representative protostome bilaterian. An alignment of all homeodomains (with accession numbers) is presented in Additional data file 1. The number of homeodomains we identified in the human and fruit fly genomes is comparable to a recent analysis of bilaterian homeodomains that identified 102 in *Drosophila *and 257 in humans [48]. The present analysis includes fewer homeodomains from human and fruit fly because we eliminated hypothetical or computationally predicted homeodomains that introduced new gaps or extended existing gaps in the alignment. Like the aforementioned analysis, we treated individual homeodomains from multi-homeodomain genes as separate taxa in our phylogenetic analysis - lower case letters appended to the gene name distinguish different homeodomains that derive from a single protein.

Because the human and *Drosophila *genomes are still in the process of being annotated, and because our criteria for homeodomain inclusion were stringent, this dataset cannot be considered exhaustive. However, most sequences excluded from this study represent rapidly evolving and highly divergent sequences that would not have a significant bearing on the conclusions. The *Nematostella *dataset consists of first-pass predictions from a draft-quality genomic sequence. It is possible that a number of *Nematostella *homeodomains may have been missed, and it is also possible that homeodomains from one or more pseudogenes have been included. Nevertheless, these data are more than sufficient for the purpose of the analyses performed here: to obtain a qualitatively accurate assessment of the homeobox-gene complement present in the cnidarian-bilaterian ancestor.

### Overall tree topologies and classification of animal homeodomains

**Figure 3:**
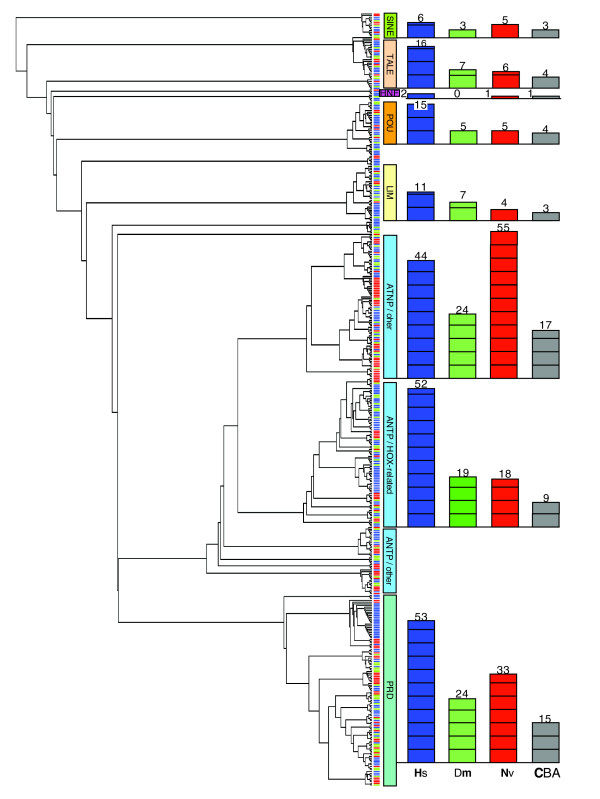
Phylogenetic relationships among homedomains from *Nematostella *(red lines), human (blue lines), and fruitfly (green lines) determined by neighbor-joining [95]. Gene names are not provided in this condensed version of the tree, which is intended to convey an overview of the homeodomain radiation in metazoans. A fully labeled version of this tree is provided in Additional data file 2. All homeodomain classes that are known to be shared among cnidarians and bilaterians are indicated by colored bars (ANTP, HNF, LIM, POU, PRD, SINE, and TALE). Histograms to the right of the tree indicate the number of sequences from each species that fall within a given class (Hs, *Homo sapiens*; Dm, *Drosophila melanogaster*; Nv, *Nematostella vectensis*). The gray bars on the histograms provide a conservative estimate for the size of each homeodomain class in the cnidarian-bilaterian ancestor (CBA). The homeodomain tallies shown here are based solely on the phylogenetic analyses performed in this study. Additional data sources, cited in the text, would lead us to adjust the tallies for *Nematostella *and the CBA slightly upward.

The homeodomain phylogeny produced by Bayesian analysis agrees substantially with the phylogeny produced by neighbor-joining (fully labeled neighbor-joining and Bayesian phylogenies are contained in Additional data files 2 and 3, respectively; Figure 3 depicts the neighbor-joining topology without individual gene names). Both trees recover nearly all of the accepted bilaterian homeodomain families with high statistical support. Throughout this paper, we emphasize phylogenetic inferences that are supported by both methods, especially those homeodomain families that receive robust statistical support from both methods, as judged by bootstrap proportions in the neighbor-joining analysis (BP) and log-likelihood values in the Bayesian analyses (LnL).

The neighbor-joining analysis supports the monophyly of the ANTP class overall, and the monophyly of a Hox-related subclass within the ANTP class. The Bayesian analysis also supports the monophyly of the Hox-related subclass. However, on the Bayesian tree, there is an unresolved polytomy at the base of the ANTP class that includes a number of non-ANTP class homeodomains. This polytomy could be resolved in a manner that is compatible or incompatible with the monophyly of the ANTP class. The HNF, POU, PRD, and SINE classes appear monophyletic on both neighbor-joining and Bayesian trees. The CUT, LIM, and ZF classes do not appear monophyletic on either the neighbor-joining or Bayesian trees (Additional data files 2 and 3).

The Bayesian and neighbor-joining trees agree on the class-level relationships of 126 out of 130 of the *Nematostella *homeodomains (96.2%). According to both trees, 72 *Nematostella *homeodomains belong to the ANTP class, one to the HNF class, four to the LIM class, five to the POU class, 33 to the PRD class, five to the SINE class, and six to the TALE class (Table 1). This represents the first report of cnidarian HNF, LIM and TALE homeodomains. Four of the *Nematostella *homeodomains group with different classes on the Bayesian and neighbor-joining trees. None of *Nematostella *sequences groups with bilaterian homeodomains of the CUT class, the PROS class, or the ZF class. However, in a subsequent search of predicted *Nematostella *genes, we were able to identify a single protein that exhibits significant similarity to bilaterian CUT genes. The extensive intermingling of homeodomains from *Nematostella*, human, and fly on the phylogeny (Figure 3) reveals that the ANTP, CUT, LIM, POU, PRD, SINE, and TALE classes had undergone substantial radiations prior to the split between Cnidaria and Bilateria.

### ANTP class

#### Hox-related subclass

Genes from the Hox-related subclass have played a prominent role in the evolution and diversification of the primary body axis in animals [22,39,49,50]. The phylogenetic analyses indicate 52 Hox-related homeodomains in human, 19 in fruit fly, and 18 in *Nematostella*. All 89 of these genes constitute a monophyletic group on both Bayesian and neighbor-joining trees (Additional data files 2 and 3). Within this large clade of Hox related genes, we can identify 15 distinct monophyletic families (Additional data file 1; Table 1). On both the Bayesian and neighbor-joining trees, eight of these families appear to have *Nematostella *representatives: CDX, EVX, EXEX, GBX, GSX, HOX1, MOX, and ROUGH. Previous studies have reported CDX, EVX, GBX, GSX, HOX1, and MOX genes in cnidarians [17,37-40,51], but EXEX and ROUGH homeodomains have not previously been identified in this phylum. According to the neighbor-joining tree, the HOX2 family may also be represented in *Nematostella*, which would be consistent with previously published homeodomain phylogenies that have identified putative anterior Hox genes (HOX1 and HOX2 families) in the Cnidaria [17,38,39,51]. No *Nematostella *sequences group with the HOX3, HOX4, HOX5, HOX6-8, or HOX9-13 families. The apparent absence of 'central' Hox genes (HOX4-HOX8) in cnidarians, has been a consistent finding of recent phylogenetic analyses, but these same studies have supported the existence of 'posterior' Hox genes in cnidarians (HOX9-HOX13) [17,38,39,51]. For example, in published neighbor-joining and maximum likelihood analyses, the *Nematostella *homeodomains *anthox1 *and *anthox1a *have grouped with posterior Hox genes in bilaterians [17,22,38]. In the present analysis, these same homeodomain sequences (known as NVHD099 and NVHD106) either fall basal to a clade containing both posterior and central genes (Bayes), or they fall basal to a clade comprising all the central Hox genes (neighbor-joining).

While previous studies have reported multiple Hox-related ANTP genes from individual cnidarian species, including EVX, MOX, GSX, and Hox genes [17,37-40,51], the present study is unique in terms of its scope and the thoroughness with which the Hox-related homeodomains have been sampled from a single cnidarian genome. No previous study has reported as many as 18 Hox-related genes from a member of this phylum. The inclusion of numerous additional sequences has resulted in the identification of previously unreported families (EXEX and ROUGH), and it has caused us to question the previously hypothesized relationships of NVHD099 and NVHD106. The current analysis does not support the designation of these genes as posterior Hox genes. The Bayes tree suggests an interesting alternative hypothesis - that these two *Nematostella *homeodomains could be direct descendants of the common ancestor of central and posterior Hox genes. This could explain the apparent absence of central Hox genes without the need to invoke gene loss [12,52]. More detailed phylogenetic and gene linkage studies of *Nematostella *and other basal metazoan lineages may help to elucidate the early evolution of Hox-related genes.

#### Other ANTP class families

We identified 122 ANTP class homeodomains that fall outside the Hox-related clade: 44 from human, 24 from fruit fly, and 54 from sea anemone. Of these 122 homeodomains, 98 can be classified into one of 21 different gene families (Additional data file 1; Table 1). According to both trees, *Nematostella *appears to possess representatives from 17 of these 21 families (Additional data files 2 to 3). Single *Nematostella *homeodomains group with each of the following families: DLX, HHEX, HMX, LBX, MSX, NK-1 (slouch), NK-3, NK-6, NK-7, and TLX. The statistical support for these groupings is very robust, with neighbor-joining bootstrap proportions and Bayesian log-likelihood values in excess of 0.88 in all cases. Multiple *Nematostella *homeodomains group with each of the following families: EMX (two sequences), EMXLX (two sequences), HLX (seven sequences), MSLX (two sequences), NK-2 (five sequences), and VAX (two sequences). Two *Nematostella *homeodomains also group with the predicted *Drosophila *homeodomain CG13424 in what appears to be a very ancient, but not formally recognized family of ANTP-class homeodomains. While CG13424 appears missing in the human genome, two CG13424-related proteins have been described in another deuterostome, the appendicularian urochordate *Oikopleura dioica *[53]. None of the *Nematostella *homeodomains groups with the following four families on either of the trees: BARH, BARX, BSH, and EN. Twenty-two of the *Nematostella *sequences could not be assigned to a specific family. The results presented here, bolstered by previous studies that have reported BARX, DLX, EMX, HHEX, MSX, NK-2, and TLX genes from other cnidarians [39,44,54-56], make it clear that the ANTP class had radiated extensively prior to the cnidarian-bilaterian split.

### CUT class

The genes of the Cut class [3], also known as the Cut superclass [6,57], typically encode two different types of DNA-binding domains: homeodomains as well as cut domains [58-60]. Cut domains are roughly 80 amino acids long, and they are typically located upstream of the homeodomain [6]. Cut proteins may possess only a single cut domain (as in Onecut), two cut domains (as in the SATB genes), or three cut domains, (as in the *Drosophila *gene Cut [58]). Genes of the Compass family lack a Cut domain altogether, but they are placed within this class on the basis of their shared possession with the SATB genes of a conserved COMPASS domain at the amino terminus [6]. The Cut class is believed to be monophyletic on the basis of the shared possession of the cut domain (in all but the Compass family) and on the basis of phylogenetic analyses of homeodomain and cut domain sequences [59].

On both the neighbor-joining and Bayesian phylogenies produced here, each of the four previously recognized subgroups of Cut genes appears monophyletic (COMPASS, CUTL, ONECUT, and SATB [6]). However, the class as a whole does not appear monophyletic on either tree. On the Bayesian tree, the ONECUT family appears closely related to the CUTL family, but the COMPASS and SATB families emerge as independent lineages. On the neighbor-joining tree, all four Cut families emerge as distantly related independent lineages. Clearly, when a broad representation of homeodomain proteins is considered, phylogenetic analysis of the homeodomain does not support the monophyly of the Cut class. On the Bayesian tree, none of the *Nematostella *homeodomains groups with Cut class homeodomains. On the neighbor-joining tree, two *Nematostella *homeodomains do group with the SATB genes in a weakly supported clade (BP = 0.14). The phylogenetic analyses clearly imply that the CUT class had not diversified prior to the cnidarian-bilaterian split.

However, an independent analysis suggests that the primordial CUT gene did originate prior to the split between Cnidaria and Bilateria, and that this gene most resembled the ONECUT family, as previously predicted [6]. We have identified a single putative CUT gene in the *Nematostella *genome by searching the database of predicted genes at StellaBase [46,47] for CUT domains (query conditions: Protein Family Name: CUT; E-value threshold: 1e-6). The single gene returned by this search (StellaBase ID: 14839) encodes both a Cut domain and a homeodomain. The top 50 hits in a BLASTp search of the non-redundant protein database using this protein as the query are all CUT class proteins, specifically members of the ONECUT family.

### HNF class

The HNF class is a small class of homeodomain proteins that was erected to accommodate HNF1, a liver-specific transcription factor (hepatic nuclear factor) with a highly atypical homeodomain [61]. The homeodomains of the HNF class are unusual in that they possess a large number of extra residues between helix 2 and helix 3 [6]. So far, this homeodomain class has not been reported outside of vertebrates. On both the neighbor-joining and Bayesian trees, there is robust support for a clade uniting two human HNF homeodomains (HNF1a, HNF1b) with the *Nematostella *sequence NVHD070 (Additional data files 1 to 3). No *Drosophila *sequence groups with this HNF clade.

### LIM class

The LIM homeobox genes are characterized by two protein-binding zinc fingers called LIM domains, which are located upstream of the homeodomain [62]. LIM homeodomain proteins are widely implicated in neural patterning throughout the animal kingdom [62,63]. Recently, a LIM-domain containing gene was reported in *Nematostella *[64], but this gene does not encode a homeodomain. No LIM-class homeodomains have yet been described for the phylum Cnidaria.

The phylogenetic analysis presented here identifies 11 LIM homeodomains in human, 7 in fruit fly, and 4 in *Nematostella *(Table 1; Additional data files 1 to 3). The LIM class is divided into six distinct groups: APTEROUS, ISLET, LIN-11, LHX3/4, LHX6/8, and LMX [62]. In our trees, all six of these groups represent discrete clades. Here, we refer to the LIN-11 class as the LHX1/5 group based on the names of the human and fruit fly genes that belong to it. If we limit the membership of the LIM class to these six groups, then the LIM class appears paraphyletic on the neighbor-joining and Bayesian trees (Additional data files 2 and 3). In both the Bayesian and neighbor-joining trees, a number of zinc-finger homeodomains disrupt the monophyly of the LIM class. On both neighbor-joining and Bayesian trees, the ISLET, LIM1/5, and LHX6/8 clades each contain a single *Nematostella *gene. The *Nematostella *homeodomain NVHD055 appears as the sister to a clade comprising the LHX1/5 and LHX3/4 families on both the neighbor-joining tree and the Bayes tree.

### POU class

POU genes are characterized by an approximately 75 amino acid DNA binding domain upstream of the homeodomain. During development, their expression is known to be spatially and temporally restricted, and they have been implicated in cell-fate determination, early embryonic development and neuronal determination [65]. The POU class comprises six different families [65]. POU I genes have been reported from non-Bilateria such as sponges [66] and cnidarians (D Jacobs, personal communication). POU IV and VI genes have also been described in a cnidarian [67].

*Nematostella *has five putative POU genes, including single representatives from the POU I, IV, and VI families, and potentially two representatives from the POU III family (Additional data files 1 to 4). Class II and class V genes appear lacking in *Nematostella*. *Drosophila*, like *Nematostella*, is missing a class V gene, which suggests that this class may be a vertebrate invention. On the other hand, *Drosophila *is missing a class I gene. Its absence in the fruit fly and presence in sea anemone and human suggests a possible gene loss in the line leading to *Drosophila*. We can surmise that at least four POU homeodomains were present in the cnidarian-bilaterian ancestor, including single representatives of classes I, III, IV, and VI. Class II may be a bilaterian invention.

### PRD class

Both the neighbor-joining and Bayes trees support the monophyly of a PRD clade comprising 53 human homeodomains, 24 fruit fly homeodomains, and 33 *Nematostella *homeodomains (Additional data files 1 to 3). A previous phylogenetic analysis of PRD homeodomains delineated the following distinct evolutionary lineages: Al, Anf (HESX1), Arix, Cart1 (ALX3/4), Ceh10, Gsc, Mix, Og12 (SHOX), Otp, Otx, Pax3/7, Pax4/6, Prx, Ptx, Rx, Siamois (DUX), and Unc4 [5]. All but two of these lineages appear monophyletic on both Bayesian and neighbor-joining trees - the Bayesian tree does not support the monophyly of the ALX3/4 and AL families. Three additional homeodomain families reside within the PRD radiation on the Bayesian and neighbor-joining trees, bringing the total number of PRD families to 20 - the DMBX, HB (Homeobrain), and REPO families are each represented in both *Nematostella *and the Bilateria, and they cannot be subsumed within the 17 PRD lineages that were defined previously [5,68].

On both the Bayesian and neighbor-joining trees, 15 of the 20 PRD families harbor *Nematostella *sequences, including several families not previously reported in the Cnidaria: AL, ALX, CEH-10, DMBX, DUX, GSX, HB, OTP, OTX, PAX3/7, PAX4/6, PTX, REPO, RX and UNC4 (Additional data files 1 to 3; Table 1). *Nematostella *appears to lack a representative from the ARIX and PRX families, which are found in fruit fly and human, and from the ANF and MIX families, which are found only in human. The fruit fly appears to lack representatives of the ALX, DMBX, and DUX families, all of which are represented in the human and sea anemone. Likewise, three of the groups found in fruit fly and sea anemone appear to lack a human representative: HB, REPO, and UNC4.

The phylogenetic analyses suggest that the cnidarian-bilaterian ancestor may have possessed representatives of 15 PRD homeodomain families. The ANF, ARIX and PRX families may have originated within the Bilateria. Three PRD families may have been lost in the line leading to *Drosophila *(ALX, DMBX, DUX), while three different PRD families may have been lost in the line leading to human (HB, REPO, and UNC4).

The DUX family is home to several human genes with double and triple homeodomains. Interestingly, three closely linked *Nematostella *homeodomains group with the human DUX homeodomains. These *Nematostella *homeodomains may be part of the same locus. If all three homeodomains are expressed as part of a single protein, it would be the first reported triple-homeodomain gene in a cnidarian. However, the statistical support for the branches uniting human DUX homeodomains with these potential *Nematostella *DUX homeodomains is low (BP = 0.21; LnL = 0.35), and the existence of a single transcript comprising all three homeodomains has not been demonstrated experimentally in *Nematostella*, so this homology assignment must be regarded as tentative pending additional evidence. Also, the two most closely linked of these putative DUX homeoboxes (DuxA and DuxC) are extremely similar at the nucleotide level, both within the homeobox itself and in an intron that interrupts the homeobox. This is a region of the assembly rife with repeated sequence, a condition that would be consistent with either a very recent tandem duplication or a false duplication caused by an error in the assembly. A molecular analysis of this region will be required to verify the assembly.

### SINE class

SINE class genes (for example, *Drosophila sine oculis *and vertebrate *six *genes) possess a highly distinctive homeodomain in addition to a conserved *Six*/*so *domain, 120 amino acids in length, that is located upstream of the homeodomain. Three families are recognized (SIX1/2, SIX3/6, and SIX4/5) [6]. All three families have been reported from the Cnidaria previously [45,69]. A single SIX1/2 class gene has also been recovered from sponges [45].

We identified six SINE homeodomains in human, three in fly, and five in *Nematostella*. Both the neighbor-joining and Bayesian trees support the monophyly of the SINE class and the monophyly of each of its constituent families. On both trees, *Nematostella *homeodomain NVHD073 groups with the SIX1/2 family, NVHD128 groups with the SIX3/6 family, and NVHD030 groups with the SIX4/5 family. Two other *Nematostella *homeodomains (NVHD061 and NVHD093) fall within the SINE class, but their exact phylogenetic positions differ between trees. All five of these predicted homeodomain sequences are located in close proximity to predicted *Six*/*so *domains (data not shown). The findings of this study and previous studies make it very clear that the SINE family had expanded to encompass three distinct members prior to the cnidarian-bilaterian split [45,69].

### TALE class

Homeodomains of the TALE (three amino acid loop extension) class are characterized by the possession of three extra amino acids in the loop between helix 1 and helix 2 of the homeodomain [6]. TALE homeodomains have been recovered from bilaterian animals, plants, and fungi [6,70]. We identified 16 TALE class homeodomains from human, 7 from *Drosophila*, and 6 from *Nematostella*. This appears to be the first report of TALE class homeodomains in a non-bilaterian metazoan. On both the neighbor-joining and Bayesian trees, the four recognized families of TALE homeodomains appear monophyletic: IRX, MEIS, PBX, and TGIF [6]. All four families are represented in the *Nematostella *genome. On both trees, *Nematostella *homeodomain NVHD108 groups with the IRX class, NVHD107 groups with the MEIS class, NVHD040 groups with the PBX class, and NVHD149 groups with the TGIF class. Two *Nematostella *homeodomain sequences (NVHD036 and NVHD143) fall within the TALE radiation, but their precise position differs between the neighbor-joining and Bayesian trees. Five of the six of the *Nematostella *TALE homeodomains contain three extra amino acids in the same position as in human and fly. The sixth, NVHD036 actually contains four extra amino acids in this location. In five of six Nematostella TALE homeodomains, the first extra residue is a histidine, just as in bilaterians.

### ZF class

Proteins of the ZF class are known to encode as many as 4 homeodomains and 17 zinc fingers [6]. The homeodomain sequences are highly divergent. It has been suggested that the large number of DNA-binding domains present per protein might reduce the evolutionary constraints operating on the evolution of each individual DNA-binding domain [6]. Presumably, the shared possession of zinc fingers reflects a shared common ancestry of ZF class homeodomains. However, neither of the homeodomain phylogenies supports the monophyly of this class. A few well supported ZF homeodomain families can be recognized on both trees, but none of these families includes a *Nematostella *representative (Additional data files 1 to 3). At this time, it appears possible that this homeodomain class is specific to bilaterians.

### Introns

The presence or absence of introns and their location relative to the homeodomain may provide evidence regarding homeodomain phylogeny. However, in the Bilateria, this trait appears evolutionarily labile, and so the phylogenetic utility of homeodomain introns may be compromised by rampant homoplasy [3]. In the Bilateria, homeobox genes from all 10 classes may possess introns that interrupt the homeodomain, and these introns have been found to occur at over 20 different positions within the homeodomain (Additional data file 1) [3].

In contrast to the Bilateria, in *Nematostella*, the presence and location of homeodomain-interrupting introns appears much more evolutionarily stable (Additional data file 1). In *Nematostella*, only the HNF, PRD, and TALE class exhibit introns within the homeodomain. Furthermore, the location of introns within the homeodomain is highly consistent. Of the 130 *Nematostella *homeodomains included in this study, 38 are interrupted by introns (Additional data file 1). Three *Nematostella *homeodomains are interrupted by two introns each (NVHD170 of the HNF class plus NVHD107 and NVHD036, both of the TALE class). The overwhelming majority of these introns (33/41) are located at nucleotide position 139 of the canonical 180-nucleotide homeobox. Nearly all members of the PRD class in *Nematostella *(31/33) contain an intron at this location. The only PRD class homeodomains to lack an intron at this location are sequences that cannot be assigned to a particular family (NVHD031 and NVHD052).

The possession of an intron at the identical location in nearly all *Nematostella *PRD homeodomains reinforces the conclusion that the PRD class is monophyletic. One *Nematostella *homeodomain of uncertain class affinities (NVHD088) also exhibits an intron in the same location as 31 of the PRD sequences. This sequence is nested within the PRD radiation in the Bayesian tree, but it falls outside of the PRD radiation in the neighbor-joining tree. This sequence may in fact be a member of the PRD class.

Three homeodomains from the TALE class and the lone representative of the HNF class are also interrupted by introns in *Nematostella*. The TALE class homeodomain of NVHD040 (PBX) is interrupted by a single intron at nucleotide position 133 of its 189-nucleotide homeobox. The homeoboxes of two other TALE class members, NVHD107 (MEIS) and NVHD036 are each interrupted by two introns. Likewise, the homeodomain of NVHD070 (HNF class) is interrupted by two introns. Two homeodomains whose class membership is ambiguous (NVHD045 and NVHD007) are interrupted by a single intron at nucleotide position 133 of their 189-nucleotide homeoboxes, just as in the TALE class homeodomain NVHD040.

The intron situation in *Nematostella *contrasts markedly with that in *Drosophila *and humans. These bilaterian organisms possess many more PRD-class homeodomains that lack introns, many more non-PRD-class homeodomains that contain introns, and the position of introns within the homeodomain is highly variable (Additional data file 1). These data suggest that an intron was introduced at position 139 of the homeobox in the ancestral Paired homeodomain. Subsequently, after the divergence of Cnidaria and Bilateria, there has been a greater constraint on loss or gain of homeodomain introns within the Cnidaria. Additional analyses are needed to determine whether this constraint on intron gain or loss is specific to the homeodomain superfamily or whether it might be a general feature of cnidarian genomes. If intron location proves to be a particularly stable trait in many cnidarian genes, then the Cnidaria may prove extremely valuable for elucidating the early evolution of metazoan gene families.

## Discussion

It is clear that a major radiation of homeobox genes occurred prior to the split between the Cnidaria and Bilateria. As expected, human homeodomains substantially outnumber fruit fly or anemone homeodomains. Typically, each homeodomain family contains two to three times as many human representatives as fruit fly representatives. This partly reflects the large scale genomic duplications that are known to have occurred in the history of the deuterostomes [71,72]. However, it is surprising that the sea anemone, a morphologically simple animal and an outgroup to the Bilateria, would possess substantially more homeodomains than the fruit fly (130 versus 97). This result may be attributed to three factors. The sea anemone inherited a large complement of homeodomains from the cnidarian-bilaterian ancestor, the fruit fly has experienced some apparent homeodomain loss, and the anemone has experienced numerous homeodomain duplications after its divergence from the Bilateria.

### Homeodomain families in the cnidarian-bilaterian ancestor

How many homeodomains were present in the cnidarian-bilaterian ancestor? If we infer that every homeodomain family shared by *Nematostella *and the Bilateria was represented by a single ancestral sequence in their common ancestor, an inference consistent with the phylogenetic analyses, then this ancestor possessed at least 56 homeodomains (Table 1; Figure 3). The phylogenetic affinities of some *Nematostella *homeodomains are less well supported than others, and it is likely that a few homeodomains are misidentified here. However, our phylogenetic reconstruction seeks to strike a balance between two types of error: misidentifying particular *Nematostella *homeodomains as orthologs of particular bilaterian homeodomains; and failing to recognize true orthology between particular homeodomains in *Nematostella *and bilaterians. The latter error forces us to assume evolutionary events (gene duplications) that never actually occurred. The estimate given here for the homeodomain complement of the cnidarian-bilaterian ancestor almost certainly represents an underestimate because more cnidarian homeodomains will be found in the future, and because many sequences that were included in this analysis could not be placed unambiguously into specific families.

Some of these difficult-to-classify sequences may derive directly from ancestral genes that were present in the cnidarian-bilaterian ancestor. For example, on the Bayesian tree, NVHD099 and NVHD106 appear as the sister group to a large clade containing central and posterior Hox families. These cnidarian genes could be directly descended from a single central/posterior ancestral sequence in the cnidarian-bilaterian ancestor. Taking this into account, our estimate for the number of homeoboxes in the genome of the cnidarian-bilaterian ancestor could plausibly be increased from 56 to 57.

Two other factors could cause us to underestimate the number of homeodomains present in the cnidarian-bilaterian ancestor. In some instances, homeodomains derived from a common ancestor may have diverged so substantially in the three lineages represented in this study that they can no longer be recognized as members of the same family. In other instances, gene loss in either *Nematostella *or the two bilaterian systems could hide the fact that a particular homeodomain was present in the cnidarian-bilaterian ancestor.

### Homeodomain families unique to Bilateria

In our dataset, 17 different gene families shared by human and fruit fly appear to be lacking in *Nematostella*. Five of these are Hox-related homeodomains: HOX3, HOX4, HOX5, HOX6-8, and HOX9-13. Other ANTP class genes that are shared by the bilaterians but missing from *Nematostella *are BARX, BSH, and EN. *Nematostella *also appears to lack two CUT families that are shared between human and fruit fly (CUTL and ONECUT), three LIM families (AP, LHX3/4, and LMX), one POU family (POU2), two PRD families (ARIX and PRX), and one ZF family (ZFH2). Additional gene surveys may identify some of these 'missing' genes in the genome of *Nematostella *or other Cnidaria (for example, the identification of a likely CUT gene in *Nematostella *that was discussed above). However, if the absence of particular homeodomain families in Cnidaria can be confirmed, then we may one day attribute the evolution of certain bilaterian traits to the origin and diversification of these key developmental regulators. Homeodomain proteins found in Bilateria but apparently lacking in Cnidaria (such as central Hox genes, EN, and BSH) are implicated in the development of important bilaterian body plan features, including segmentation, paired appendages, and brains.

### Homeodomain loss in human and fruit fly?

Recent expressed sequence tag (EST) studies on cnidarians have demonstrated that gene loss has been rampant in some bilaterian model systems, particularly the model protostomes *Drosophila *and *Caenorhabditis elegans *[73,74]. In this study, we observed several homeodomain families that are present in *Nematostella *but appear to be missing in either human or fruit fly. Six homeodomain families are present in the human and the anemone but appear to be missing from the fly (ALX, DMBX, DUX, HNF1, POU1, and VAX), while eight homeodomain families are present in the fly and the anemone but appear to be missing from the human (CG13424, EMXLX, HB, MSXLX, NK7, REPO, ROUGH and UNC4).

The conclusion that these genes have been lost is not significantly affected by the exclusion of computationally predicted homeodomains that introduced new gaps or extended existing gaps in the alignment - several such sequences were included in the Nam and Nei study [48] but left out of the present study. We performed a neighbor-joining analysis on the 257 human and 102 fly sequences from the Nam and Nei study (not shown). Except for a single human sequence, a partial-homeodomain that grouped with the genes of the *Unc4 *family, none of the other families identified in this study as missing in the human or fruit fly was present in the larger dataset [4,48]. The partial *Unc4 *homeodomain was removed from our analysis because it introduced gaps into the alignment. It is possible that this *Unc4*-like sequence is a pseudogene.

**Figure 4:**
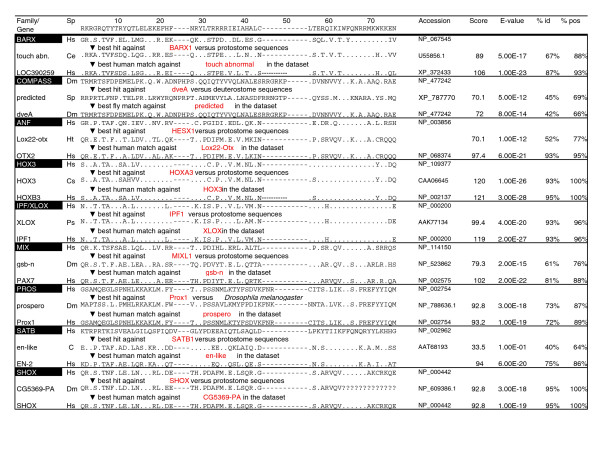
Reciprocal protostome versus deuterostome BLAST searches. Reciprocal BLAST searches were used to identify protostome representatives of missing fly homeodomains and deuterostome representatives of missing human homeodomains. Human homeodomains representing the ANF, BARX, HOX3, IPF/XLOX, MIX, PROX, SATB, and SHOX families were used as queries for BLASTp searches of protostome entries in the non-redundant (NR) protein database. The top hit was then BLASTed back against our dataset. Similarly, the fruit fly dveA homeodomain (COMPASS family) was used as a query to search deuterstome proteins. The top hit was then blasted back against our dataset. The initial query sequence and the top hits in each BLASTp search are aligned to the *Drosophila Antennapedia *homeodomain. The BLASTp scores and E-values are shown, as are the percentage of amino acid 'identities' (% id) and 'positives' (% pos). Species abbreviations are as follows: Bf, *Branchiostoma floridae*; C, *Capitella *species; Ce, *Caenorhabditis elegans*; Cs, *Cupiennius salei*; Dm, *Drosophila melanogaster*; Hs, *Homo sapiens*; Ht, *Helobdella triserialis*; Ps, *Phascolion strombi*; Sp, *Strongylocentrotus purpuratus*.

If homeodomain families are being lost (or modified beyond recognition) over the course of animal evolution, then some families that appear unique to human or fruit fly in our dataset may in fact be shared among protostomes and deuterostomes. By utilizing BLAST searches and consulting previously published studies, we were able to demonstrate that HOX3, COMPASS, IPF, SHOX, and PROS are distributed across both protostomes and deuterostomes, despite the fact that, in our dataset, they are missing from either the human or the fly. For example, while none of the *Drosophila *homeodomains group with the vertebrate HOX3 homeodomains on the phylogenies, a BLAST of the human HoxA3 homeodomain against protostome sequences identifies a clear HOX3 homeodomain in the spider *Cupiennius *(Figure 4). Furthermore, while not supported by our analyses, there is evidence from other phylogenetic studies, gene expression, and gene linkage that *Drosophila *zen1, zen2, and bcd are actually derived members of the HOX3 family [75-77]. The IPF/XLOX family also appears to be missing from *Drosophila*, but XLOX genes have been reported from a number of protostome animals, including sipunculans and annelids [78-80]. Among protostomes, the best match to the human IPF homeodomain is the XLOX homeodomain from the sipunculan worm *Phascolion strombus *(Figure 4) [78]. The COMPASS family appears to be missing from human, but BLASTp of the *Drosophila *dveA homeodomain against all deuterostome sequences detected a clear homolog in the sea urchin *Strongylocentrotus *(Figure 4). Our bioinformatic survey of *Drosophila *homeodomains failed to retrieve a representative of the SHOX family or the PROS class. However, a BLASTp search with human SHOX homeodomain against protostome sequences identified a predicted protein in *Drosophila *with near perfect resemblance over the first 47 amino acids (45/47 identities). The predicted protein appears to be missing residues 48 to 60 of the homeodomain. This may be an error in the annotation, which would explain why we failed to include this putative homeodomain sequence in our dataset. A BLASTp search with human *Prox1 *against protostome sequences identified the *Drosophila prospero *homeodomain (Figure 4).

### Why does *Nematostella *outnumber *Drosophila*?

The results presented here suggest that the fruit fly has lost some homeodomain sequences that were present in the cnidarian-bilaterian ancestor, including HNF1, VAX, POU1, ALX3/4, DMBX, and DUX (Tables 1 and 2). This is not entirely unexpected given that widespread gene loss in *Drosophila *has been revealed previously by comparison of cnidarian and bilaterian ESTs [73,74]. However, the number of homeodomains that appear missing from the human genome slightly exceeds the number missing in *Drosophila *(Table 2; eight versus six, respectively), so any loss of homeodomain sequences from *Drosophila *does not appear extreme.

The invention of novel homeodomains in the lineage leading to *Nematostella *contributes more to the sea anemone's excess over the fruit fly than does the number of missing homeodomains in *Drosophila*. After all, the loss of 6 homeodomains in *Drosophila *is more than offset by the presence of 18 homeodomains that are present in fruit fly and human but absent in the anemone (Table 2). The phylogenetic analyses, in concert with gene linkage data [81] (unpublished results), indicate that the lineage leading to *Nematostella *has experienced tandem duplication of many homeobox families, including MOX, HOX1, HOX2, and OTX. Particularly within the ANTP class and the PRD class, there are extensive homeodomain radiations that appear unique to the sea anemone (Figure 3; Additional data files 2 and 3). Kusserow and co-workers [82] revealed similar *Nematostella*-specific radiations within the Wnt gene superfamily.

It is important to note that the combination of recent tandem duplication and polymorphism creates an analytical challenge for the assembly. Polymorphism may cause the assembly to overestimate the number of distinct homeoboxes in the *Nematostella *genome by mistaking different alleles for distinct loci. This possibility can be ruled out when the regions flanking the sequences in question are highly distinctive. However, recent tandem duplications can juxtapose closely related homeoboxes surrounded by highly similar flanking sequences. After careful examination of the regions flanking three pairs of related homeoboxes, we cannot absolutely rule out the possibility that these may be false gene duplications due to assembly errors: NVHD003/064, NVHD007/045, and NVHD102/043. Furthermore, the three candidate DUX homeodomains NVHD005, NVHD011, and NVHD038 reside in a particularly complex region featuring lots of repetitive sequence. Experimental evidence will be required to validate the assembly in these regions.

## Conclusion

If the evolution of homeobox genes has been critical to the evolution of morphological diversity in animals [6,19-24], then it is important to establish when particular homeobox genes first appeared in metazoan evolution. The results presented here provide the first glance at a nearly complete homeodomain complement in a non-bilaterian metazoan. These data allow us to infer the condition found in the common ancestor of Cnidaria and Bilateria. All of the major homeobox classes (ANTP, LIM, POU, PRD, SINE, and TALE) must have undergone a significant radiation prior to the evolutionary split between Cnidaria and Bilateria. Conservatively, we estimate that 56 distinct homeodomain families were represented in the cnidarian-bilaterian ancestor. Seventeen specific homeodomain families present in fly and human were found to be absent in *Nematostella*, and these may represent bilaterian inventions. Surprisingly, the sea anemone *Nematostella*, a simple non-bilaterian animal, possesses far more homeodomains than the fruit fly (131 versus 97). The sea anemone's numerical advantage over *Drosophila *can be attributed mostly to the origin of new homeoboxes in the cnidarian lineage.

The results presented here emphasize that there is no simple relationship between the complexity of gene families and the complexity of organisms. Cnidarians have fewer distinct body regions and about five-fold fewer distinctive cell types than arthropods [29], yet *Nematostella *has substantially more homeobox genes than *Drosophila*. Measures of morphological complexity, such as the number of cell types, may not be tightly correlated with gene number [83]. More complex organisms may possess fewer genes than simpler organisms, but each gene of the more complex organism may be deployed in a greater number of distinct spatiotemporal contexts [83]. Global comparisons of gene number, and even comparisons within particular gene families, may, therefore, prove insufficient to illuminate the genomic causes of organismal complexity. Future functional studies should be directed at understanding the consequences of particular gene radiations for particular organismal lineages. Genome-wide phylogenetic analyses such as this will be required to identify such gene radiations.

We must caution that all of the results described here are based on phylogenetic analysis of an undoubtedly incomplete dataset of homeodomain sequences. The ongoing annotation of the human, fruit fly, and *Nematostella *genomes will allow us to build on this dataset, thereby improving our understanding. In addition, the sequencing of additional bilaterian and basal metazoan genomes will allow us to consult more taxonomic sources so that our inferences about higher taxa are based on more data points. Complementary data types may also prove useful, including other protein domains where appropriate (for example, cut domains, six/so domains, LIM domains, paired domains, and so on), and data on genomic linkage. Finally, as our datasets steadily increase in size, the development of more rapid and more sophisticated computational methods for the analysis and representation of gene family evolution may yield insights that are not currently attainable.

## Materials and methods

### Retrieval of *Nematostella *homeodomains

We assembled the publicly available *Nematostella *shotgun traces generated by the Joint Genome Institute using the Phusion assembler [84]. The traces may be obtained through the Trace Archive v3.0 at the National Center for Biotechnology Information, USA [85]. The Phusion program generated the following statistics regarding the assembly (contig-bases: 360061553 bases; contig-N50: 10888 bases; contig-count: 81401; coverage: 7.6X; genome-size: 400 to 450 Mb, estimated from word count distribution; scaffold-size: 381073596 bases; scaffold-N50: 49588 base; scaffold-count: 50021; heterozygosity: approximately 1 single nucleotide polymorphism in 250 bases.) This assembly is searchable at the StellaBase website [46,47].

A set of deuterostome homeodomains downloaded from the Homeodomain Resource [86] were BLASTed against the assembled *Nematostella *genome. Four kilobase genomic sequences surrounding matches that showed significant similarity to the deuterostome homeodomains (TBLASTN E values < 0.001) were extracted from the genome. These segments were run through the GENSCAN program [87]. Homeodomain motifs were then extracted from predicted proteins. In cases where no gene was predicted, the genomic segments were translated in six frames and the homeodomains corresponding to the BLAST hit were extracted. The homeodomains and the genomic sequences from which the homeodomains were derived have been submitted to GenBank.

### Retrieval of human and fly homeodomains

The complete set of proteins of *H. sapiens *and *D. melanogaster *were downloaded from NCBI's RefSeq database in FASTA format (2004-10-14) [88,89]. These sequences were screened using the homeodomain profile from PFAM (2004-08-20) [90] and the *hmmsearch *program from the *HMMer *software suite [91]. A custom Perl script was used to extract the homeodomain sequences from the FASTA files according to the hits reported by *hmmsearch *(Additional data file 4). Each homeodomain from multi-homeodomain genes was treated as a separate taxon. The human, *Drosophila*, and *Nematostella *sequences were aligned by eye to the alignment of human homeodomains published by Banerjee-Basu and Baxevanis [4] using the GeneDoc software [92]. To avoid long-branch artifacts associated with derived sequences and spurious predictions, homeodomains from RefSeq sequences that introduced new gaps into the alignment and had not been experimentally verified were discarded.

### Phylogenetic analysis

Bayesian analysis was performed using MrBayes version 3.1.2-MPI [93]. Fixed rate models were estimated by MrBayes (aamodelpr = mix). The Markov chain Monte Carlo search was run for 10,000,000 generations with trees being sampled every 100 and printed every 1,000 generations. By default, MrBayes performs two simultaneous, completely independent analyses starting from different random trees (Nruns = 2). These 2 runs generated 10,000 trees each. These 2 treefiles were meshed and the first 4,000 trees were discarded as 'burnin'. The Consense program from PHYLIP [94] was used to build a 'Majority rule (extended)' tree from the remaining 16,000 trees. A neighbor-joining [95] analysis was performed using PHYLIP (version 3.6.1) [94]. The Dayhoff PAM matrix was used to generate the distance matrix. Support for clades on the neighbor-joining tree was assessed by 1,000-replicates of bootstrap [96]. The phylogenetic dataset is available as a text file in NEXUS format (Additional data file 5).

### Intron analysis

The location of *Nematostella *introns was determined by aligning homeobox sequences to their corresponding genomic regions using the GenBank submission tool, Sequin [97]. Splice junctions were confirmed to conform to the GT-AG rule by Sequin's submission validation process. *Drosophila *and human introns were aligned to their corresponding genomes with the alignment tool BLAT [98]. Intron locations were chosen for each homeodomain from the best hit for each search.

### BLAST searches to identify missing bilaterian genes

BLAST searches were used to identify possible protostome representatives of homeodomain families that were represented in our data only by human sequences (HOX3, IPF/XLOX, BARX, SATB, ANF, MIX, and SHOX). The human homeodomain sequences were used to query the non-redundant (NR) protein database using BLASTp. The BLAST searches were performed through the NCBI web site using the Entrez query terms "protostomia[ORGN]". The top hit was then BLASTed back against human protein sequences for missing *Drosophila *sequences, and "deuterostomia[ORGN]" for missing human sequences. The top hit and those hits that shared an E-value within the same order of magnitude as the top hit were BLASTed back against our three-species homeodomain dataset. If the top hit (or a hit that shared an E-value within the same order of magnitude as the top hit) was a member of the missing family, that sequence was considered to be orthologous.

## Additional data files

The following additional data are available with the online version of this paper. Additional data file 1 is an alignment of all homeodomains included in the phylogenetic analysis. Accession numbers and phylogenetic affinities are provided for each sequence, including the degree of statistical support for each homeodomain's phylogenetic position on both the neighbor-joining and Bayesian trees. Additional data file 2 is a neighbor-joining phylogeny depicting the relationships among 455 distinct homeodomain sequences (130 from *Nematostella*, 97 from *Drosophila*, and 228 from human). Additional data file 3 is a Bayesian phylogeny depicting the relationships among the same 455 homeodomain sequences. Additional data file 4 is a Perl script that was used to parse BLAST reports and extract homeodomains from corresponding FASTA files. Additional data file 5 is the phylogenetic dataset used in this study in nexus format.

## Supplementary Material

Additional data file 1 Accession numbers and phylogenetic affinities are provided for each sequence, including the degree of statistical support for each homeodomain's phylogenetic position on both the neighbor-joining and Bayesian trees. Click here for file

Additional data file 2 Neighbor-joining phylogeny depicting the relationships among 455 distinct homeodomain sequences (130 from *Nematostella*, 97 from *Drosophila*, and 228 from human). Click here for file

Additional data file 3 Bayesian phylogeny depicting the relationships among the same 455 homeodomain sequences. Click here for file

Additional data file 4 Perl script that was used to parse BLAST reports and extract homeodomains from corresponding FASTA files. Click here for file

Additional data file 5 Phylogenetic dataset used in this study in nexus format. Click here for file

## Figures and Tables

**Table 1:** Number of homeodomain proteins by class, family, and species

	Hs	Dm	Nv	CBA
**ANTP class/Hox-related**				
CDX*	3	1	1	1
EVX*	2	1	1	1
EXEX*	1	1	1	1
GBX*	2	1	1	1
GSX*	2	1	1	1
HOX1*	3	1	2	1
HOX2*	2	1	3	1
HOX3*	3	3	0	0
HOX4*	4	1	0	0
HOX5*	3	1	0	0
HOX6-8*	8	3	0	0
HOX9-13*	16	1	0	0
IPF*	1	**0**^†^	0	0
MOX*	2	1	4	1
ROUGH*	0	1	1	1
Unknown family	0	1	3	n/a
**Total**	**52**	**19**	**18**	**9**
				
**ANTP class/other**				
BARH*	2	2	0	0
BARX	2	0	0	0
BSH*	1	1	0	0
CG13424*	0	1	2	1
DLX*	6	1	1	1
EMX*	2	2	2	1
EMXLX*	0	1	2	1
EN*	2	1	0	0
HHEX*	1	1	1	1
HLX*	2	2	7	1
HMX*	3	1	1	1
LBX*	2	2	1	1
MSX*	2	1	1	1
MSXLX*	0	1	2	1
NK1*	1	1	1	1
NK2*	7	2	5	1
NK3*	2	1	1	1
NK6*	2	1	1	1
NK7*	0	1	1	1
TLX*	3	1	1	1
VAX*	1	0	2	1
Unknown family	3	0	22	n/a
**Total**	**44**	**24**	**54**	**17**
				
**CUT class**				
COMPASS	0	2	0	0
CUTL*	2	1	0	0
ONECUT*	3	1	0	0
SATB	2	0	0	0
**Total**	**7**	**4**	**0**	**0**
				
**HNF**				
HNF1/2*	**2**	**0**	**1**	**1**
				
**LIM class**				
AP*	2	1	0	0
ISLET*	2	1	1	1
LHX1/5*	2	1	1	1
LXH3/4*	2	1	0	0
LHX6/8*	2	1	1	1
LMX*	1	2	0	0
Unknown family	0	0	1	n/a
**Total**	**11**	**7**	**4**	**3**
				
**POU class**				
POU1*	1	0	1	1
POU2*	3	2	0	0
POU3*	4	1	2	1
POU4*	3	1	1	1
POU5	2	0	0	0
POU6*	2	1	1	1
**Total**	**15**	**5**	**5**	**4**
				
**PRD**				
AL*	1	2	1	1
ALX*	3	0	1	1
ANF	1	0	0	0
ARIX*	2	1	0	0
CEH10*	2	2	1	1
DMBX*	1	0	6	1
DUX*	20	0	3	1
GSC*	2	1	1	1
HB*	0	1	1	1
MIX	1	0	0	0
OTP*	1	1	1	1
OTX*	3	1	3	1
PAX3/7*	2	3	2	1
PAX4/6*	3	4	2	1
PRX*	2	1	0	0
PTX*	3	1	1	1
REPO*	0	1	1	1
RX*	2	1	1	1
SHOX	2	0	0	0
UNC4*	0	2	1	1
Unknown family	2	2	7	n/a
**Total**	**53**	**24**	**33**	**15**
				
**PROS class**				
PROS	1	0	0	0
**Total**	1	0	0	0
				
**SINE class**				
SIX1/2*	2	1	1	1
SIX3/6*	2	1	1	1
SIX4/5*	2	1	2	1
Unknown family	0	0	1	n/a
**Total**	**6**	**3**	**5**	**3**
				
**TALE**				
IRX*	7	3	1	1
MEIS*	3	1	1	1
PBX*	4	1	1	1
TGIF*	1	2	1	1
Unknown family	1	0	2	n/a
**TOTAL**	**16**	**7**	**6**	**4**
				
**ZF class**				
ZFHX2	2	0	0	0
ZFH4	2	0	0	0
ZHX	5	0	0	0
zfh1	0	1	0	0
zfh2*	1	1	0	0
Unknown family	1	0	0	n/a
**Total**	**11**	**2**	**0**	**0**
				
**Unknown class**				
**Total**	**10**	**2**	**4**	**n/a**

*Counted as a shared family in Table 2. ^†^Absence of IPF in *Drosophila *is due to secondary loss. CBA, cnidarian-bilaterian ancestor; Hs, *Homo sapiens*; Dm, *Drosophila melanogaster*; Nv, *Nematostella vectensis*.

**Table 2:** Homeodomain families shared by two species but missing from the third (sorted by species and homeodomain class)

Class	Total number of shared families	Shared families missing in Hs	Shared families missing in Dm	Shared families missing in Nv
HOX	15*	1	1^†^	6^†^
other	20	4	1	3
CUT	2	0	0	2
HNF	1	0	1	0
LIM	6	0	0	3
POU	5	0	1	1
PRD	17	3	3	2
PROS	0	0	0	0
SINE	3	0	0	0
TALE	4	0	0	0
ZF	1	0	0	1
Total	73	8	7	18

*In the HOX class, IPF/XLOX is counted as a shared class because, even though it is not found in *Drosophila*, its presence in other protostome animals makes clear that its absence in the fruit fly is due to a secondary loss. ^†^Secondary loss of IPF/XLOX is known to have occurred in *Drosophila*. This gene is found in other protostome animals and so, while it is scored as missing from *Drosophila*, it is also regarded as a shared family among bilaterians that is missing in *Nematostella*. Hs, *Homo sapiens*; Dm, *Drosophila melanogaster*; Nv, *Nematostella vectensis*.
