# Supplementary material for: The cnidarian-bilaterian ancestor possessed at least 56 homeoboxes: evidence from the starlet sea anemone, Nematostella vectensis
Source: Genome Biol. 2006 Jul 24;7(7):R64. doi: 10.1186/gb-2006-7-7-r64 (PMC1779571; doi:10.1186/gb-2006-7-7-r64)
Supplement: Additional data file 1 — Accession numbers and phylogenetic affinities are provided for each sequence, including the degree of statistical support for each homeodomain's phylogenetic position on both the neighbor-joining and Bayesian trees. [file gb-2006-7-7-r64-S1.pdf]

ANTP CLASS

Gene	Sp	Sequence (10 20 30 40 50 60 70)	Accession	NJ BP	Bayes LnL	Intron Pos
		RKRGRQTYTRYQTLELEKEFHF----NRYLTRRRRIEIAHALC-----------LTERQIKIWFQNRRMKWKKEN				
		**ANTP CLASS / HOX-RELATED**				
		**CDX / Caudal family**		0.32	0.44	
CDX1	Hs	KDKY.VV..DH.R........Y----S..I.I..KS.L.AN.G-----------....V........A.ER.V.	NP_001795			130
CDX2	Hs	KDKY.VV..DH.R........Y----S..I.I..KA.L.AT.G-----------.S...V........A.ER.I.	NP_001256			133
CDX4	Hs	KEKY.VV..DH.R........C----...I.IQ.KS.L.VN.G-----------.S...V........A.ER.MI	NP_005184			130
cad	Dm	KDKY.VV..DF.R......YCT----S..I.I..KS.L.QT.S-----------.S...V........A.ER.Q.	NP_476954			
**NVHD065**	Nv	.S.A.TA..AS.Q.......LY----S..I..T..K.L.NT.D-----------.S.KH..........K..TD	DQ301955			
		**Evx / even-skipped family**		0.99	0.84	
EVX1	Hs	MR.Y.TAF..E.IAR.....YR----EN.VS.P..C.L.A..N-----------.P.TT..V........D.RQK	NP_001980			142
EVX2	Hs	VR.Y.TAF..E.IAR.....YR----EN.VS.P..C.L.A..N-----------.P.TT..V........D.RQR	AAA52414			142
Eve	Dm	VR.Y.TAF..D.LGR.....YK----EN.VS.P..C.L.AQ.N-----------.P.ST..V........D.RQR	NP_523670			
**EVX (109)**	Nv	TR.Y.TAF..E.LKR.....MR----EN.VS.T..C.L.N..N-----------.S.TT...........S.RRR	DQ206338			
		**Exex / HlxB9 family**		0.93	0.99	
HLXB9	Hs	CR.P.TAF.SQ.L....HQ.K.----.K..S.PK.F.V.TS.M-----------...T.V............RSK	NP_005506			130
exex	Dm	TR.P.TAF.SQ.L.....Q.KQ----.K..S.PK.F.V.SG.M-----------.S.T.V............RSK	NP_648164			
**HLXB9 (069)**	Nv	HR.P.TAFSSH.L.A..RQ.QL----HK....PQ.Y.L.TS.M..............T.V............RC.	DQ206324			
		**Gbx / Unplugged family**		0.94	0.99	
GBX1	Hs	SR.R.TAF.SE.L........C----KK..SLTE.SQ.....K-----------.S.V.V........A...RIK	AAA36002			
GBX2	Hs	SR.R.TAF.SE.L........C----KK..SLTE.SQ.....K-----------.S.V.V........A...RVK	NP_001476			
unplugged	Dm	SR.R.TAF.SE.L....R...A----KK..SLTE.SQ..TS.K-----------.S.V.V........A...RVK	NP_477146			133
**GBX (018)**	Nv	.R.K.TAF.SK.L.Q..R...N----KK.VSLEE.SV..TN.N-----------...V.V........A...RVR	DQ206305			
		**Gsx / Intermediate Neuroblasts Defective family**		0.80	0.96	
GSH1	Hs	S..M.TAF.ST.L....R..AS----.M..S.L......TY.N-----------.S.K.V........V.H...G	NP_663632			
GSH2	Hs	G..M.TAF.ST.L....R..SS----.M..S.L......TY.N-----------.S.K.V........V.H...G	NP_573574			
ind	Dm	S..I.TAF.ST.L....R..SH----.A..S.L......NR.R-----------.S.K.V........V.Q.KGG	NP_996087			
**GSX (130)**	Nv	S..I.TA..SM.L.......SQ----....S.L...Q..AL.D-----------.S.K.V........V....DK	AAD39349			
		**Hox1 family**		0.10	0.26	
HOXA1	Hs	PNAV.TNF.TK.LT.......----.K....A..V...AS.Q-----------.N.T.V..........Q..RE	NP_005513			
HOXB1	Hs	PSGL.TNF.TR.LT.......----.K..S.A..V...AT.E-----------.N.T.V..........Q..RE	NP_002135			
HOXD1	Hs	SSAI.TNFSTK.LT.......----.K....A......NC.H-----------.NDT.V..........Q..RE	NP_078777			
lab	Dm	NNS..TNF.NK.LT.......----......A......NT.Q-----------.N.T.V..........Q..RV	NP_476613			130
**anthox6 (012)**	Nv	SQKK.F.F.QR.LV.......----SK....T......TT.K-----------...M..............R.F	DQ206301			
**NVHD060**	Nv	SDKN.TI.STR.LV.......Y----....C.P......QS.E-----------...K.V...............	DQ206321			
		**Hox2 family**		0.98	0.99	
HOXA2	Hs	SR.L.TA..NT.L........----.K..C.P..V...AL.D-----------.....V.V........H.RQT	NP_006726			
HOXB2	Hs	AR.L.TA..NT.L........----.K..C.P..V...AL.D-----------.....V.V........H.RQT	NP_002136			
pb	Dm	PR.L.TA..NT.L........----.K..C.P......AS.D-----------.....V.V........H.RQT	NP_996162			130
**anthox7 (029)**	Nv	T..Y.TS..NR.L........Y----.K..CGT..R.L.N.MK-----------.....V.V........L..DE	DQ206311	0.21	n/a	
**anthox8a (006)**	Nv	S..H.TS..NK.L........----.K..CSS..R..SK..Q-----------.....V.............DE	DQ206299	0.21	n/a	
**anthox8b (171)**	Nv	S..H.TS..NK.L........----.K..CSS..R..SK..Q-----------.....V.............DE	DQ315389	0.21	n/a	
		**Hox3 family** (known in insects; probable Dm homologs=zen1,zen2,bcd)		1.00	0.98	
HOXA3	Hs	S..A.TA..SA.LV.......----....C.P..V.M.NL.N-----------..................Y..DQ	NP_109377			
HOXB3	Hs	S..A.TA..SA.LV.......----....C.P..V.M.NL.N-----------.S................Y..DQ	NP_002137			
HOXD3	Hs	S..V.TA..SA.LV.......----....C.P..V.M.NL.N-----------..................Y..DQ	NP_008829			
bcd***	Dm	PR.T.T.F.SS.IA...QH.LQ----G....AP.LADLSAK.A-----------.GTA.V....K...RRH.IQS	NP_476825	***	***	
zen***	Dm	L..S.TAF.SV.LV...N..KS----.M..Y.T......QR.S-----------.C...V..........F..DI	NP_476793	***	***	
zen2***	Dm	S..S.TAFSSL.LI...R...L----.K..A.T.....SQR.A-----------.....V..........L..ST	NP_476794	***	***	

ANTP CLASS (continued)

Gene	Sp	10 20 30 40 50 60 70<br>RKRGRQTYTRYQTLELEKEFHF----NRYLTRRRRIEIAHALC-----------LTERQIKIWFQNRRMKWKKEN	Accession	NJ BP	Bayes LnL	Intron Pos
		ANTP CLASS / HOX-RELATED (continued)				
		**Hox4 family**		0.55	0.75	
HOXA4	Hs	P..S.TA...Q.V.........----..............T..-----------.S...V.............DH	NP_002132			
HOXB4	Hs	P..S.TA...Q.V........Y----.........V.......-----------.S.................DH	NP_076920			
HOXC4	Hs	P..S.AA...Q.V........Y----..............S..-----------.S.................DH	NP_055435			
HOXD4	Hs	P..S.TA...Q.V.........----..............T..-----------.S.................DH	NP_055436			
Dfd	Dm	P..Q.TA...H.I........Y----..............T.V-----------.S.................D.	NP_477201			
		**Hox5 family** (monophyly not supported on Bayesian tree)		0.19	n/a	
HOXA5	Hs	G..A.TA...............----.................-----------.S.................D.	NP_061975			
HOXB5	Hs	G..A.TA...............----.................-----------.S.................D.	NP_002138			
HOXC5	Hs	G..S.TS...............----.............NN..-----------.N.................DS	NP_061826			
Scr	Dm	T..Q.TS...............----.................-----------....................H	NP_996164			
		**Hox6-8 family**		0.42	0.28	
HOXA6	Hs	GR....................----.............N...-----------.....................	NP_076919			
HOXB6	Hs	GR...................Y----.................-----------....................S	NP_061825			
HOXC6	Hs	.R....I.S.............----.............N...-----------....................S	NP_004494			
HOXA7	Hs	......................----.................-----------....................H	NP_008827			
HOXB7	Hs	.....................Y----..............T..-----------.....................	NP_004493			
HOXB8	Hs	.R......S...........L.----.P....K....VS...G-----------.....V...............	NP_076921			
HOXC8	Hs	.RS.....S...........L.----.P....K....VS...G-----------.....V...............	NP_073149			
HOXD8	Hs	.R......S.F.........L.----.P....K....VS...A-----------.....V...............	NP_062458			
Antp	Dm	......................----.................-----------.....................	NP_996172			
Ubx	Dm	.R...................T----.H.........M.....-----------................L...I	NP_996219			
Abd-A	Dm	.R........F...........----.H...............-----------................L...L	NP_476693			
		**Hox9-13 family**		0.66	0.44	
HOXA9	Hs	TRKK.CP..KH.........L.----.M....D..Y.V.RL.N-----------.....V..........M..I.	NP_002133			
HOXB9	Hs	SRKK.CP..K..........L.----.M....D..H.V.RL.N-----------.S...V..........M..M.	NP_076922			
HOXC9	Hs	TRKK.CP..K..........L.----.M....D..Y.V.RV.N-----------.....V..........M..M.	NP_008828			
HOXD9	Hs	TRKK.CP..K..........L.----.M....D..Y.V.RI.N-----------.....V..........M..MS	NP_055028			
HOXA10	Hs	GRKK.CP..KH.........L.----.M....E..L..SRSVH-----------..D..V..........L..M.	NP_061824			
HOXC10	Hs	GRKK.CP..KH.........L.----.M....E..L..SKTIN-----------..D..V..........L..M.	NP_059105			
HOXD10	Hs	GRKK.CP..KH.........L.----.M....E..L..SKSVN-----------..D..V..........L..MS	NP_002139			
HOXA11	Hs	TRKK.CP..K..IR...R..F.----SV.INKEK.LQLSRM.N-----------..D..V..........E..I.	NP_005514			
HOXC11	Hs	SRKK.CP.SKF.IR...R..F.----.V.INKEK.LQLSRM.N-----------..D..V..........E..L.	NP_055027			
HOXD11	Hs	TRKK.CP..K..IR...R..F.----.V.INKEK.LQLSRM.N-----------..D..V..........E..LS	NP_067015			
HOXC12	Hs	SRKK.KP.SKL.LA...G..LV----.EFI..Q..R.LSDR.N-----------.SDQ.V..........K.RLL	NP_776272			
HOXD12	Hs	ARKK.KP..KQ.IA...N..LV----.EFIN.QK.K.LSNR.N-----------.SDQ.V..........K.RVV	NP_067016			
HOXA13	Hs	GRKK.VP..KV.LK...R.YAT----.KFI.KDK.RR.SATTN-----------.S...VT.......V.E..VI	NP_000513			
HOXB13	Hs	GRKK.IP.SKG.LR...R.YAA----.KFI.KDK.RK.SA.TS-----------.S....T.......V.E..VL	NP_006352			
HOXC13	Hs	GRKK.VP..KV.LK....YAA----SKFI.KEK.RR.SATTN-----------.S...VT.......V.E..VV	NP_059106			
HOXD13	Hs	GRKK.VP..KL.LK...N.YAI----.KFINKDK.RR.SA.TN-----------.S...VT.......V.D..IV	NP_000514			
Abd-B	Dm	VRKK.KP.SKF.........L.----.A.VSKQK.W.L.RN.Q-----------.....V..........N..NS	NP_996220			130
		**IPF family** (=Xlox; found in protostomes other than Dm)		n/a	n/a	
IPF1	Hs	N..T.TA...A.L.......L.----.K.IS.P..V.L.VM.N-----------....H...............E	NP_000200			

Gene	Sp	Sequence (10 20 30 40 50 60 70)	Accession	NJ BP	Bayes LnL	Intron Pos
**ANTP CLASS** (continued)						
		RKRGRQTYTRYQTLELEKEFHF----NRYLTRRRRIEIAHALC-----------LTERQIKIWFQNRRMKWKKEN				
ANTP CLASS / HOX-RELATED (continued)						
**Mox / Buttonless family**				0.95	1.00	
MOX1	Hs	ARKE.TAF.KE.LR...A..AH----HN....L..Y...VN.D-----------.S...V.V..........RVK	NP_004518			130
MOX2	Hs	PRKE.TAF.KE.IR...A..AH----HN....L..Y...VN.D-----------....V.V..........RVK	NP_005915			133
btn	Dm	NRKE.TAFSKT.LKQ..A..CY----SN....L..Y...V..E-----------....V.V.........C.RIK	NP_732768			
**MOXa (034)**	Nv	KRKE.TAF.TH.LR...N..TR----.N....L..Y...VS.D-----------....V.V..........RVK	DQ206313			
**MOXb (110)**	Nv	KRKE.TAF.KH.IQ...N..QR----.N....L..Y...VS.D-----------....V.V..........RVK	DQ206339			
**MOXc (019)**	Nv	KRKE.TAFSKH.LQ...N..IR----.N....L..Y...VS.D-----------....V.V..........RVK	DQ206306			
**MOXd (119)**	Nv	KRKE.TVFSK..LT...R..CR----.N....L..Y...VS.D-----------.G...V.V..........RGR	DQ206342			
**Rough family**				0.99	1.00	
ro	Dm	QR.Q.T.FSTE...R..V...R----.E.IS.S..F.L.ET.R-----------...T..........A.D.RIE	NP_733173			
**RO (126)**	Nv	PR.Q.T.F.SE...K..L...Q----.E.I..S..F.L.AC.N-----------...T.V........A.D.RLE	DQ206293			
**HOX-RELATED SUBCLASS; family membership ambiguous**				n/a	n/a	
Ftz	Dm	S..T...................----...I......D..N..S-----------.S..............S..DR	NP_477498			
**anthox1a (099)**	Nv	KH.K.MA...I.L.........----T....KE..T.M.RM.D-----------....V.............D.	DQ206337			
**anthox1 (106)**	Nv	G..K.TA...K.L.........----.HF..KE..S.M.SQ.N-----------....V.............C.	DQ206287			
**NVHD117**	Nv	NHAP.SAF.TV.Q..I....LY----DH.VS.V.....VM..D-----------.S.K.VRT........L.R.A	DQ206290	0.10	n/a	
ANTP CLASS / OTHER						
**BARH family**				0.97	1.00	
BARHL1	Hs	PRKA.TAF.DH.LAQ..RS.ER----QK..SVQD.M.L.AS.N-----------..DT.V.T.Y....T...RQT	NP_064448			160
BARHL2	Hs	PRKA.TAFSDH.LNQ..RS.ER----QK..SVQD.MDL.A..N-----------..DT.V.T.Y....T...RQT	NP_064447			160
B-H1	Dm	QRKA.TAF.DH.LQT...S.ER----QK..SVQE.Q.L..K.D-----------.SDC.V.T.Y....T...RQT	NP_523387			160
B-H2	Dm	QRKA.TAF.DH.LQT...S.ER----QK..SVQD.M.L.NK.E-----------.SDC.V.T.Y....T...RQT	NP_523386			160
**BARX family**				0.99	0.89	
BARX1	Hs	GR.S.TVF.EL.LMG...R.EK----QK..STPD..DL.ES.G-----------.SQL.V.T.Y.........IV	NP_067545			91
BARX2	Hs	PR.S.TIF.EL.LMG...K.QK----QK..STPD.LDL.QS.G-----------..QL.V.T.Y.........MV	NP_003649			91
**BSH family**				0.99	1.00	
LOC390259	Hs	.RKA.TVFSDS.LSG...R.EI----Q...STPE.V.L.T..S-----------.S.T.V.T........H..QL	XP_372433			
bsh	Dm	.RKA.TVFSDP.LSG...R.EG----Q...STPE.V.L.T..G-----------.S.T.V.T........H..QL	NP_995728			
**CG13424 family**				0.66	0.90	
CG13424	Dm	K..P.TAFSAA.IKA..T..ER----GK..SVAK.TAL.KQ.Q-----------...T..........T...RKY	NP_611491			
**NVHD021**	Nv	K..T.TAF.NT.IR...A..QK----.K...IT..A.L.NS.E-----------.SDT..........T.L.RKL	DQ206308			
**NVHD027**	Nv	K..P.TAF.NE.IKD..A..QK----SK..SVS..M.L.NS.S-----------...T..........T...RKM	DQ206263			
**DLX family**				0.98	1.00	
DLX1	Hs	IRKP.TI.SSL.LQA.NRR.QQ----TQ..ALPE.A.L.AS.G-----------..QT.V......K.S.F..LM	NP 835221			130
DLX2	Hs	VRKP.TI.SSF.LAA.QRR.QK----TQ..ALPE.A.L.AS.G-----------..QT.V........S.F..MW	NP 004396			133
DLX3	Hs	VRKP.TI.SS..LAA.QRR.QK----AQ..ALPE.A.L.AQ.G-----------..QT.V........S.F..LY	NP 005211			130
DLX4	Hs	LRKP.TI.SSL.LQH.NQR.QH----TQ..ALPE.AQL.AQ.G-----------..QT.V......K.S.Y..LL	NP 001925			133
DLX5	Hs	VRKP.TI.SSF.LAA.QRR.QK----TQ..ALPE.A.L.AS.G-----------..QT.V......K.S.I..IM	NP 005212			127
DLX6	Hs	IRKP.TI.SSL.LQA.NHR.QQ----TQ..ALPE.A.L.AS.G-----------..QT.V......K.S.F..LL	XP 376652			130
Dll	Dm	MRKP.TI.SSL.LQQ.NRR.QR----TQ..ALPE.A.L.AS.G-----------..QT.V........S.Y..MM	NP 523857			130
**DLX (095)**	Nv	IRKP.TI.SSF.LR..N.R.IK----TQ..ALPE.ADL.AY.G-----------..QT.V........S.F..TL	DQ206283			

ANTP CLASS (continued)

Gene	Sp	10 20 30 40 50 60 70<br>RKRGRQTYTRYQTLELEKEFHF----NRYLTRRRRIEIAHALC-----------LTERQIKIWFQNRRMKWKKEN	Accession	NJ BP	Bayes LnL	Intron Pos
		**ANTP CLASS / OTHER**				
		**EMX family**		0.92	1.00	
EMX1	Hs	P..I.TAFSPS.L.R..RA.EK----.H.VVGAE.KQL.GS.S-----------.S.T.V.V......T.Y.RQK	NP 004088			130
EMX2	Hs	P..I.TAFSPS.L.R..HA.EK----.H.VVGAE.KQL..S.S-----------..T.V.V......T.F.RQK	NP 004089			130
ems	Dm	P..I.TAFSPS.L.K..HA.ES----.Q.VVGAE.KAL.QN.N-----------.S.T.V.V......T.H.RMQ	NP 731868			
E5	Dm	P..V.TAFSPT.L.K..HA.EG----.H.VVGAE.KQL.QG.S-----------..T.V.V......T.H.RMQ	NP 524825			133
**EMXa (013)**	Nv	P..I.TAF.PT.L.H..NA.EK----.H.IVGTE.KQL.SY.N-----------.S.T...V......T...RQQ	DQ206302			
**EMXb (104)**	Nv	VR.V.TAF.PF.L.C..TS.DK----.H.VVGTE.KQL.SY.K-----------.S.T.V.V......T...RQA	DQ206286			
		**EMXLX family**		0.88	0.61	
CG18599	Dm	N..V.TIF.PE.LEC..A..ER----QQ.MVGPE.LYL..T.K-----------..A.V.V......I..R.HH	NP 610261			
**EMXLX (080)**	Nv	P..I.TIF.PE.LER.....ER----QQ.MVGAE.HYL.AS.N-----------..T.V.V......I..R.QK	DQ206278			
**NVHD077**	Nv	P..V.TIFSPD.LDR..R..DN----QQ.IVGTE.FYL.TE.G-----------..T.V.V......I..R.Q.	DQ206329			
		**EN family**		0.97	0.99	
EN1	Hs	D..P.TAFTAE.LQR.KA..QA----...I.EQ..QTL.QE.S-----------.N.S........K.A.I..AT	NM 001426			
EN2	Hs	D..P.TAFTAE.LQR.KA..QT----.....EQ..QSL.QE.S-----------.N.S........K.A.I..AT	NM 001427			
en	Dm	E..P.TAFSSE.LAR.KR..NE----.....E...QQLSSE.G-----------.N.A........K.A.I..ST	NM 078976			
		**HHEX family**		0.94	0.88	
HHEX	Hs	..G.QVRFSND..I....K.ET----QK..SPPE.KRL.KM.Q-----------.S...V.T......A..RRLK	NP 002720			133
CG7056	Dm	..G.QIRF.SQ..KN..AR.AS----SK..SPEE.RHL.LQ.K-----------..D..V.T......A..RRAN	NP 650938			
**HHEX (048)**	Nv	K.G.QVRFSNE..M....I.ET----QK..SPPE.KQLSKV.G-----------.S...V.T......A..RRFK	DQ206317			
		**HLX family**		0.07	0.24	
HLX1	Hs	.SWS.AVFSNL.RKG...R.EI----QK.V.KPD.KQL.AM.G-----------..DA.V.V.........RHSK	NP 068777			127
FLJ16139	Hs	GILR.AVFSED.RKA...M.QK----QK.ISKTD.KKL.IN.G-----------.K.S.V...........RNSK	NP 001004329			133
CG12361	Dm	GMMR.AVFSDS.RKG...R.QQ----QK.ISKPD.KKL.ER.G-----------.KDS.V...........RNSK	NP 647677			28;130
H2.0	Dm	.SWS.AVFSNL.RKG..IQ.QQ----QK.I.KPD.RKL.AR.N-----------..DA.V.V.........RHTR	NP 523488			142
**HLXa (082)**	Nv	PP.P.PVFSHF.RQH..NY.LS----KK..NKTE.LK..E..G-----------.S.N.V........V..RA.V	DQ206331			
**HLXb (015)**	Nv	.PWS.PVF.QL.RRG...R.QV----QK.I.KHD.YQL.AM.G-----------.S.T.V.V......T..RQDM	DQ206303			
**HLXc (092)**	Nv	.PWT.AVFSNL.RKG...R.QV----QK...KAD.HQL.SM.G-----------..DN.V.V.........RQDA	DQ206333			
**HLXd (121)**	Nv	KQKQ.PLFGQK.IQR..E..VC----EK.ISKS..SAL.AEID-----------..DA.VRT......TR.R..E	DQ206343			
**HLXe (094)**	Nv	KPKQ.PLFSHN.IQR.....KE----EK...ESK.A.LSKD.G-----------M..T.V.T......T..R..L	DQ206282			
**HLXf (049)**	Nv	PTKL.PLFSKT.LSR.....SG----.K...ESK.EQLSKD.G-----------M..T.V.T......T..R.KK	DQ206318			
**HLXg (035)**	Nv	.PKL.PLFSKT.LSR.....EE----K....ETK.A.LSKD.E-----------M..T.V.T......T..R..K	DQ206314			
		**HMX family**		0.99	0.99	
HMX1	Hs	K.KT.TVFS.S.VFQ..ST.DL----K...STAE.AGL.AS.Q-----------...T.V........N...RHV	NP 061815			175
HMX2	Hs	K.KT.TVFS.S.VYQ..ST.DM----K...SSSE.ACL.SS.Q-----------...T.V.T......N...RQL	NP 005510			
LOC340784	Hs	K.KT.TVFS.S.VFQ..ST.DM----K...SSSE.AGL.AS.H-----------...T.V........N...RQL	XP 291716			
Hmx	Dm	K.KT.TVFS.A.VFQ..ST.DL----K...SSSE.AGL.AS.R-----------...T.V........N...RQL	NP 524951			
**HMX (116)**	Nv	K.KT.TVFS.S.VYQ..ST.DM----K...SSSE.AGL.SQ.H-----------...T.V........N...RQI	DQ206341			
		**LBX family**		0.95	1.00	
LBX1	Hs	.RKS.TAF.NH.IY....R.LY----QK..SPAD.DQ..QQ.G-----------..NA.VIT......A.L.R.?	NP 006553			
Loc400962	Hs	.RKS.TAF.AQ.V....RR.V.----QK..APSE.DGL.TR.G-----------.ANA.VVT......A.L.RD?	NP 001009812			
lbe	Dm	KRKS.TAF.NH.IF....R.LY----QK..SPAD.D...AS.G-----------.SNA.VIT......A.Q.RDI	NP 524435			
lbl	Dm	KRKS.TAF.NQ.IF....R.LY----QK..SPAD.D...GG.G-----------.SNA.VIT......A.L.RDM	NP 524434			130
**LBX (122)**	Nv	.RKP.TCF.NR.II...RR.MY----QK..SPSD.DD..M..G-----------.PGA..IT......A.MRRDV	DQ206291			
		**MSX family**		0.99	0.94	
MSX1	Hs	NRKP.TPF.TA.L.A..RK.RQ----KQ..SIAE.A.FSSS.S-----------...T.V........A.A.RLQ	NP 002439			
MSX2	Hs	NRKP.TPF.TS.L.A..RK.RQ----KQ..SIAE.A.FSSS.N-----------...T.V.......SA.A.RLQ	NP 002440			
Dr	Dm	NRKP.TPF.TQ.L.S...K.RE----KQ..SIAE.A.FSSS.R-----------...T.V........A.A.RLQ	NP 477324			
**MSX (142)**	Nv	NRKP.TPF.TS.L.A..RK.RQ----KQ..SIAE.A.FSAS.N-----------...T.V........A.A.RLH	DQ206296			

ANTP CLASS (continued)

Gene	Sp	10 20 30 40 50 60 70<br>RKRGRQTYTRYQTLELEKEFHF----NRYLTRRRRIEIAHALC-----------LTERQIKIWFQNRRMKWKKEN	Accession	NJ BP	Bayes LnL	Intron Pos
		ANTP CLASS / OTHER (continued)				
		**MSXLX family**		0.72	0.77	
CG15696	Dm	GRLP.IPF.PQ.LQA..NAYKE----SN..SAEDANKL.DS.E-----------..NTRV........ARERR.K	NP_650921			
**MSXLXa (046)**	Nv	SGNP.VPFSTS.LM...HKYVE----T...SGPEVA.L.NG.R-----------.S.HRV........ARE..AK	DQ206269			
**MSXLXb (066)**	Nv	DRNP.IPFSAS.LAA..AK.LD----TH..SSVEVRDLSSS.S-----------V..HRV........ARE..TK	DQ206275			
		**NK-1 (Slouch) family**		0.99	0.89	
C10orf121	Hs	PR.A.TAF.YE.LVA..NK.RA----T...SVCE.LNL.LS.S-----------...T.V........T....Q.	XP_372331			
slou	Dm	PR.A.TAF.YE.LVS..NK.KT----T...SVCE.LNL.LS.S-----------...T.V........T....Q.	NP_476657			130
**SLOU (091)**	Nv	PR.A.TAFTYE.LVA..NK.KS----T...SVCE.LNL.LS.G-----------...T.V........TNGRSRT	DQ206255			
		**NK-2 family**		0.49	0.71	
CSX	Hs	.RKP.VLFSQA.VY...RR.KQ----Q...SAPE.DQL.SV.K-----------..ST.V........Y.C.RQR	NP 032726			
LOC137814	Hs	.RKP.VLFSQA.V.A..RR.KQ----Q...SAPE.EHL.S..Q-----------..ST.V........Y.C.RQR	XP 070619			
NKX2-2	Hs	KRKR.VLFSKA..Y...RR.RQ----Q...SAPE.EHL.SLIR-----------..PT.V......H.Y.M.RAR	NP 002500			
NKX2-3	Hs	.RKP.VLFSQA.VF...RR.KQ----Q...SAPE.EHL.SS.K-----------..ST.V........Y.C.RQR	NP 660328			
NKX2-8	Hs	..KR.VLFSKA......RR.RQ----Q...SAPE.EQL.SL.R-----------..PT.V......H.Y.L.RAR	NP 055175			
TITF1	Hs	.RKR.VLFSQA.VY...RR.KQ----QK..SAPE.EHL.SMIH-----------..PT.V......H.Y.M.RQA	NP 003308			
TTF1	Hs	.RKR.VLFSQA.VY...RR.KQ----QK..SAPE.EHL.SMIH-----------..PT.V......H.Y.M.RQA	NP 003308			
tin	Dm	KRKP.VLFSQA.V....CR.RL----KK...GAE.EI..QK.N-----------.SAT.V........Y.S.RGD	NP 524433			
vnd	Dm	KRKR.VLF.KA..Y...RR.RQ----Q...SAPE.EHL.SLIR-----------..PT.V......H.Y.T.RAQ	NP 476786			
**NK2a (057)**	Nv	KRKR.ILF.KA..FI..RR.TQ----Q...SAPE.E.L.RIAN-----------..PA.V......H.Y.YR.QM	DQ206320			
**NK2b (025)**	Nv	K.KR.ILF.KS.IF...RR.RQ----QK..SANE.EQL.RIID-----------..PT.V......H.Y.F..QI	DQ206262			
**NK2c (051)**	Nv	KRKR.ILF.KS.IY...RR.RY----Q...SAPE.EQLGRMIN-----------..PT.V......H.Y.H..QA	DQ206270			
**NK2d (103)**	Nv	KRKR.VLF.KA..Y...RR.RQ----Q...SAPE.EQL.RIIN-----------..PT.V......H.Y.F..Q.	DQ206285			
**NK2e (026)**	Nv	KRKQ.VLFSKV.RKQ...K.LE----KP..SYTE.DQL.R.VN-----------..AK.V.V....K.Y.T.ERE	DQ206310			
		**NK-3 family**		0.97	0.90	
NKX3-1	Hs	Q..S.AAFSHT.VI...RK.SH----QK..SAPE.AHL.KN.K-----------...T.V........Y.T.RKQ	NP 006158			
BAPX1	Hs	K..S.AAFSHA.VF...RR.NH----Q...SGPE.ADL.AS.K-----------...T.V........Y.T.RRQ	NP 001180			
bap	Dm	K..S.AAFSHA.VF...RR.AQ----Q...SGPE.S.M.KS.R-----------...T.V........Y.T.RKQ	NP 732637			
**NK3 (098)**	Nv	...S.AAFSHQ.VF...RR.SH----QK..SGPE.ADL.A..K-----------...T.V........Y.T.RRQ	DQ206336			
		**NK-6 family**		1.00	1.00	
NKX6-1	Hs	..HT.P.FSGQ.IFA...T.EQ----TK..AGPE.ARL.YS.G-----------M..S.V.V......T..R.KH	NP 006159			139
NKX6-2	Hs	K.HS.P.FSGQ.IFA...T.EQ----TK..AGPE.ARL.YS.G-----------M..S.V.V......T..R.RH	NP 796374			139
HGTX	Dm	K.HT.P.FSGQ.IFA...T.EQ----TK..AGPE.AKL.Y..G-----------MS.S.V.V......T..R.RH	NP 652614			139
**NK6 (112)**	Nv	..HT.P.FSGH.IFA...T.EQ----TK..AGPE.ARL.YS.G-----------M..S.V.V......T..R.RH	DQ206340			
		**NK-7 family**		0.99	1.00	
Nk7.1	Dm	K.KA.T.F.GR.IF....M.EN----KK..SASE.T.M.KL.M-----------V..T.V........T....QD	NP 650357			130
**NK7 (070)**	Nv	K.KA.T.F.GR.IF....Q.EA----KK...ATE.SDM.SL.N-----------V..T.V........T....QE	DQ206276			
		**TLX family**		0.09	0.14	
TCL3	Hs	K.KP.TSF..L.IC....R..R----QK..ASAE.AAL.K..K-----------M.DA.V.T......T..RRQT	NP 005512			172
TLX2	Hs	..KP.TSFS.S.V....RR.LR----QK..ASAE.AAL.K..R-----------M.DA.V.T......T..RRQT	NP 001525			172
TLX3	Hs	..KP.TSFS.V.IC....R..R----QK..ASAE.AAL.KS.K-----------M.DA.V.T......T..RRQT	NP 066305			172
C15	Dm	..KP.TSF..I.VA....R..K----QK..ASAE.AAL.RG.K-----------M.DA.V.T......T..RRQT	NP 476873			172
**NVHD004**	Nv	K.KP.TAF.ES.IS....R.QS----QK..GSKE.S.L.GT.G-----------..DT.V???????????????	DQ206223			
NVHD024	Nv	*A.KP.TAF.ES.IS....R.QV----TEIPREQE.S.L.GT.G-----------..DT.V.T........L.RQR*	*			
		**VAX family**		0.20	0.56	
VAX2	Hs	P..T.TSF.AE.LYR..M..QR----CQ.VVG.E.T.L.RQ.N-----------.S.T.V.V......T.Q..DQ	NP 036608			130
**VAX (072)**	Nv	P..S.TSF.PA.LDR..D..RV----DM.VVGLK.MKL.ND.N-----------.S...V.V........Y.R.R	DQ206277			
**NVHD010**	Nv	...A.TAFSAE.LKK...R.QA----.H.IVGEE.QK..KD.D-----------.S.A.V.V......T.F.RDQ	DQ206300			

ANTP CLASS (continued)

Gene	Sp	10 20 30 40 50 60 70<br>RKRGRQTYTYRYQTLELEKEFHF----NRYLTRRRRIEIAHALC-----------LTERQIKIWFQNRRMKWKKEN	Accession	NJ BP	Bayes LnL	Intron Pos
		ANTP CLASS / OTHER (continued)				
		**ANTP CLASS / OTHER; ambiguous family relationships**				
NANOG	Hs	KQKT.TVFSST.LCV.NDR.QR----QK..SLQQMQ.LSNI.N-----------.SYK.V.T....Q...S.RWQ	NP_079141			130
VENTX2	Hs	AP.V.TAF.ME.VRT..GV.QH----HQ..SPLE.KRL.REMQ-----------.S.V...T........H.RQM	NP_055283			130
LOC344022	Hs	Q..V.TMFNLE.LE....V.AK----QHN.VGKK.AQL.AR.K-----------...N.VRV......V.YQ.QQ	XP_292889			133
**NVHD067**	Nv	CRKS.TVF.DL.LRV...T.SE----QK..DSTN.AKL.QI.G-----------.N.A.V.T...........K.	DQ206323			
**NVHD097**	Nv	CRKS.TVF.DL.LRV...T.SE----QK..DTSS.SKL.QI.G-----------.N.T.V.T...........ET	DQ206335			
**NVHD115**	Nv	CRKS.TVF.DL.LRV...T.SE----QK..DTSS.AKL.QI.G-----------.N.T.V.T...........ES	DQ206289			
**NVHD009**	Nv	TRPR.SFFSAD.VNQ..RV.SA----QQ.VSAKE.A...ETFN-----------M.DE.V.N........R.RKR	DQ206261			
**NVHD017**	Nv	ARNK.PVFHPDVVAQ..QV.DE----R..ISADQ.FAL.KE.N-----------M..E.V.S..H.K.TAMT.KL	DQ206304			
**NVHD023**	Nv	T.PR.SFFSAD.VNQ..RV.TA----QQ.VSAKE.A...ETFN-----------M.DD.V.N........R.RKR	DQ206309			
**NVHD032**	Nv	T.PR.SFFSAD.VNQ..RV.AA----QQ.VSAKE.A...ET.N-----------M.DD.V..........R.RKR	DQ206266			
**NVHD042**	Nv	A.PR.SFFSAD.VSQ..RV.AA----RQ.VSAKE.A...ET.N-----------M.DD.V........V.R.RKR	DQ206315			
**NVHD043**	Nv	QP.K.PVFHPDVVAR..QF.AK----R..INAEQQY.L.KEIN-----------M..E...S.LH.K.TAM.RKL	DQ206268			
**NVHD071**	Nv	T.PR.SFFSAD.VNQ..RV.AA----HQ.VSANE.A...ET.N-----------MSDD.V..........R.RKR	DQ206325			
**NVHD076**	Nv	T.PR.SFFSAD.VNQ..RV.TA----QQ.VSAKE.A...ETFN-----------M.DD.V.N........R.RKR	DQ206328			
**NVHD096**	Nv	.TTT.SFF.EH.VAC.QRV.AN----R..VCSSE.QKL.KE.N-----------M.DQ.V.T........V.RKR	DQ206334			
**NVHD102**	Nv	QP.K.PVFHPDVVAR..QF.AK----R..INAEQ.YAL.KEIN-----------M..E...S..H.K.TAM.RKL	DQ206284			
**NVHD147**	Nv	P.KN.SSFSAE.VFR...T.EL----QQ..GTKE.QQL.L..N-----------M.DN.V.T........Q.RKR	DQ206297			
**NVHD033**	Nv	SR.R.TAF.SS.LKS..EK.QE----KK...ISE.NSL.KSMH-----------..NT.V.T......T....QM	DQ206312			
**NVHD050**	Nv	KR.K.TAF.SF.LKC..DK.K.----SK...IAE.DMM.RS.Q-----------..N....T......T...R..	DQ206319			
**NVHD054**	Nv	K..H.SHFSQL.LQY.DAI.AR----QH..S.DE.TVL.G..D-----------M..L.VRN....K.YQRRNRE	DQ206272			
**NVHD081**	Nv	NP.T.THF.ER.LKY..TYYSN----G...S.DE.TVL.Q..E-----------M..L.VRN......YQR.QKQ	DQ206330			
**NVHD056**	Nv	SR.K.TAF.SS.LKY..EK.QE----KK...ISE.N.L.KSMY-----------.SDT.V.T......T....QM	DQ206274			
**NVHD041**	Nv	FR.I.TVF..D.LQK..DR.KH----DK..STQD.N.F.RS.Q-----------..PL.L.T.Y.........DM	DQ206267			
**NVHD083**	Nv	AR...TVFSAQ.LQV..RV.AG----SQ.IVGSQ.KFL.SQ.R-----------.S.T.VRV......I..R.QM	DQ206279			
**NVHD145**	Nv	KR.K.TIF.SE.LRR..E..DK----HQ..VGTE.QNL.KS.N-----------.S.T.V........I..R.D.	DQ206346			

CUT CLASS (united by the presence of a cut domain and/or CMP domain [Burglin,

Gene	Sp	10 20 30 40 50 60 70<br>RKRGRQTYTYRYQTLELEKEFHF----NRYLTRRRRIEIAHALC-----------LTERQIKIWFQNRRMKWKKEN	Accession	NJ BP	Bayes LnL	Intron Pos
		**COMPASS (CMP) family** (placement within CUT class based on possession of CMP		**0.92**	**0.25**	
dveA	Dm	TRMRTSFDPEMELPK.Q.W.ADNPHPS.QQIQTYVVQLNALESRRGRKP-----.DVNNVVY..K.A.AAQ.RAE	NP_477242			
dveB	Dm	KRNRTFIDPVTEVPK..QW.AMNTHPSHN.ILKYTEDLNTMPYRQKFPR-----.ESKNVQF..K...A.CKRLK	NP_477242			130
		**CUTL family (not monophyletic on Bayes tree)**		**0.65**	**n/a**	
CUTL1	Hs	L.KP.VVLAPEEKEA.KRAYQQ----KP.PSPKTIEDL.TQ.N-----------.KTSTVIN..H.Y.SRIRR.L	NP_001904			160
CUTL2	Hs	I.KP.VVLAPEEKEA.R.AYQL----EP.PSQQTIELLSFQ.N-----------.KTNTVIN..H.Y.SRMRR.M	NP_056082			160
ct	Dm	S.KQ.VLFSEE.KEA.RLA.AL----DP.PNVGTIEFL.NE.G-----------.AT.T.TN..H.H..RL.QQV	NP_524764			
		**ONECUT family**		**0.98**	**0.84**	
ONECUT1	Hs	P.KP.LVF.DV.RRT.HAI.KE----.KRPSKELQ.T.SQQ..-----------.ELSTVSNF.M.A.RRSLDKW	NP_004489			
ONECUT2	Hs	Q.KS.LVF.DL.RRT.FAI.KE----.KRPSKEMQ.T.SQQ.G-----------.ELTTVSNF.M.A.RRSLEKW	NP_004843			
Loc390874	Hs	P.KQRLVF.DL.RRT.IAI.KE----.KRPSKEMQVT.SQQ.G-----------.ELNTVSNF.M.A.RRCMNRW	XP_372702			
onecut	Dm	P.KPRLVF.DL.RRT.QAI.KE----TKRPSKEMQVT..RQ.G-----------.EPTTVGNF.M.A.RRSMDKW	NP_524842			64
		**SATB family**		**0.98**	**0.95**	
SATB1	Hs	KTRPRTKISVEALGILQSFIQDV---GLYPDEEAIQTLSAQLD-----------LPKYTIIKFFQNQRYYLKHHG	NP 002962			
SATB2	Hs	KPRSRTKISLEALGILQSFIHDV---GLYPDQEAIHTLSAQLD-----------LPKHTIIKFFQNQRYHVKHHG	NP_056080			

HNF CLASS

Gene	Sp	10 20 30 40 50 60 70<br>RKRGRQTYTRYQTLELEKEFHF----NRYLTRRRRIEIAHALC-----------LTERQIKIWFQNRRMKWKKEN	Accession	NJ BP	Bayes LnL	Intron Pos
**HNF CLASS**				0.98	1.00	
HNF1A	Hs	MR.N.FKWGPASQQI.FQAYER----QKNPSKEE.ETLVEECNRAECIQRGSNLV..VRVYN..A...KEEAFRQ	NP_000449			139
HNF1B	Hs	MR.N.FKWGPASQQI.YQAYDR----QKNPSKEE.EALVEECNRAECLQRGSNLV..VRVYN..A...KEEAFRQ	NP_000536			139
**HNF (170)**	Nv	SR.N.FKWGPAS.NIL.QSYEQ----Q.NPSKEE.EALVE.CNRAECEQRGFNLV..S.VYN..A...KEETFRM	DQ206332			92:139

LIM CLASS (not monophyletic on neighbor-joining or Bayes trees)

Gene	Sp	10 20 30 40 50 60 70<br>RKRGRQTYTRYQTLELEKEFHF----NRYLTRRRRIEIAHALC-----------LTERQIKIWFQNRRMKWKKEN	Accession	NJ BP	Bayes LnL	Intron Pos
**APTEROUS family**				0.97	1.00	
LHX2	Hs	T..M.TSFKHH.LRTMKSY.AI----.HNPDAKDLKQL.QKTG-----------..K.VLQV....A.A.FRRNL	NP_004780			136
LHX9	Hs	T..M.TSFKHH.LRTMKSY.AI----.HNPDAKDLKQL.QKTG-----------..K.VLQV....A.A.FRRNL	NP_001014434			136
ap	Dm	T..M.TSFKHH.LRTMKSY.AI----.HNPDAKDLKQLSQKTG-----------.PK.VLQV....A.A..RRMM	NP_724428			142
**ISLET famly**				0.99	1.00	
ISL1	Hs	TT.V.TVLNEK.LHT.RTCYAA----.PRPDALMKEQLVEMTG-----------.SP.V.RV....K.C.D..RS	NP 002193			
ISL2	Hs	TT.V.TVLNEK.LHT.RTCYAA----.PRPDALMKEQLVEMTS-----------.SP.V.RV....K.C.D..KS	NP 665804			
tup	Dm	PT.V.TVLNEK.LHT.RTCYNA----.PRPDALMKEQLVEMTS-----------.SP.V.RV....K.C.D..KT	NP 476774			
**ISL (028)**	Nv	PT.V.TVLNEK.LHT.RTCYNA----.PRPDAMMKEQLVEMTG-----------.SP.V.RV....K.C.D..KA	DQ206225			
**LHX 1/5 family**				0.94	0.99	
LIM1	Hs	.RGPGT.IKAK.LET.KAA.AA----TPKP..HI.EQL.QETG-----------.NM.V.QV......S.ERRMK	NP 005559			136
LHX5	Hs	.RGP.T.IKAK.LET.KAA.AA----TPKP..HI.EQL.QETG-----------.NM.V.QV......S.ERRMK	NP 071758			136
Lim1	Dm	.RGP.T.IKAK.LEV.KTA.NQ----TPKP..HI.EQL.KETG-----------.PM.V.QV....K.S.ERRMK	NP 572505			
**LHX1/5 (127)**	Nv	.RGP.T.IKAK.LEA.KST.AA----TPKPS.NI.EKL.QETG-----------.NM.V.QV......S.ERRLK	DQ206345			
**LHX 3/4 family**				0.94	1.00	
LHX3	Hs	A..P.T.I.AK.LET.KSAYNT----SPKPA.HV.EQLSSETG-----------.DM.VVQV......A.E.RLK	NP 055379			136
LHX4	Hs	A..P.T.I.AK.LET.KNAYKN----SPKPA.HV.EQLSSETG-----------.DM.VVQV......A.E.RLK	NP 071758			136
Lim3	Dm	N..P.T.I.AK.LET.KTAYNN----SPKPA.HV.EQLSQDTG-----------.DM.VVQV......A.E.RLK	NP 572505			157
**LHX 6/8 family**				0.65	0.77	
LHX6	Hs	A..A.TSF.AE.LQVMQAQ.AQ----DNNPDAQTLQKL.DMTG-----------.SR.V.QV....C.ARH..HT	NP_055183			40:136
LHX8	Hs	A..A.TSF.AD.LQVMQAQ.AQ----DNNPDAQTLQKL.ERTG-----------.SR.V.QV....C.ARH..HV	NP_001001933			40:136
Awh	Dm	T..V.T.F.EE.LQV.QAN.QI----DSNPDGQDLER..SVTG-----------.SK.VTQV....S.ARQ..HI	NP_523907			
**LHX 6/8 (044)**	Nv	A..V.T.F.ED.LQI.QAN.NI----DSNPDGQDLER..QLTG-----------.SK.VTQV....S.ARQ..YG	DQ206316			
**LMX family**				0.98	1.00	
LMX1B	Hs	P..P.TIL.TQ.RRAFKAS.EV----SSKPC.KV.ETL.AETG-----------.SV.VVQV....Q.A.M.KLA	NP_002307			
CG32105	Dm	P..P.TIL.SQ.RKQFKAS.DQ----SPKPC.KV.EAL.KDTG-----------.SV.VVQV....Q.A.M.KIQ	NP_729801			
CG4328	Dm	P..P.TILNTQ.RRAFKAS.EV----SPKPC.KV.ENL.KDTG-----------.SL.IVQV....Q.A.V.KIQ	NP_648567			139
**LIM CLASS / family membership ambiguous**						
**NVHD055**	Nv	S..A.TFISND.LAF.KVAYAS----SPKT.L.D.ER..KETG-----------.DM.VVQV......A.D.RLS	DQ206345			

POU CLASS

Gene	Sp	10 20 30 40 50 60 70<br>RKRGRQTYTRYQTLELEKEFHF----NRYLTRRRRIEIAHALC-----------LTERQIKIWFQNRRMKWKKEN	Accession	NJ BP	Bayes LnL	Intron Pos
**POU1 family**				0.83	1.00	
POU1F1	Hs	KRKR.T.ISIAAKDA..RH.GE----QNKPSSQEIMRM.EE.N-----------.EKEVVRV..C...QRE.RVK	NP_000297			25
**POU1 (020)**	Nv	.RKR.T.IGLAAKEA..NH.MK----QTKPSSPEIVR..DG.R-----------.DKEVVRV..C...QRE.RVK	DQ206307			
**POU2 family**				0.88	0.74	
POU2F1	Hs	.RKK.TSIETNIRVA...S.LE----.QKP.SEEITM..DQ.N-----------MEKEV.RV..C...Q.E.RIN	NP_002688			67
POU2F2	Hs	.RKK.TSIETNVRFA...S.LA----.QKP.SEEILL..EQ.H-----------MEKEV.RV..C...Q.E.RIN	NP_002689			67
POU2F3	Hs	KRKK.TSIETNIR.T...R.QD----.PKPSSEEISM..EQ.S-----------MEKEVVRV..C...Q.E.RIN	NP_055167			67
Nub	Dm	.RKK.TSIETTIRGA...A.LA----.QKP.SEEITQL.DR.S-----------MEKEVVRV..C...Q.E.RIN	NP_476659			
pdm2	Dm	.RKK.TSIETTVRTT...A.LM----.CKP.SEEISQLSER.N-----------MDKEV.RV..C...Q.E.RIN	NP_723763			

POU CLASS (continued)

Gene	Sp	10 20 30 40 50 60 70<br>RKRGRQTYTRYQTLELEKEFHF----NRYLTRRRRIEIAHALC-----------LTERQIKIWFQNRRMKWKKEN	Accession	NJ BP	Bayes LnL	Intron Pos
		**POU3 family**		0.65	0.39	
POU3F1	Hs	KRKK.TSIEVGVKGA..SHFLK----CPKPSAHEITGL.DS.Q-----------.EKEVVRV..C...Q.E.RMT	NP 002690			
POU3F2	Hs	KRKK.TSIEVSVKGA..SH.LK----CPKPSAQEITSL.DS.Q-----------.EKEVVRV..C...Q.E.RMT	NP 005595			
POU3F3	Hs	KRKK.TSIEVSVKGA..SH.LK----CPKPSAQEITNL.DS.Q-----------.EKEVVRV..C...Q.E.RMT	NP 006227			
POU3F4	Hs	KRKK.TSIEVSVKGV..THFLK----CPKPAAQEISSL.DS.Q-----------.EKEVVRV..C...Q.E.RMT	NP 000298			
Vvl	Dm	KRKK.TSIEVSVKGA..QH..K----QPKPSAQEITSL.DS.Q-----------.EKEVVRV..C...Q.E.RMT	NP 523948			
**POU3a (125)**	Nv	KRKK.TSIEVAVKGA..NH.CK----SPKP.AQEISAL.EN.G-----------.DKEVVRV..C...Q.E.RMT	DQ206344			
**POU3b (075)**	Nv	KRKR.TIIEKNVKGV..NH.EK----MPRPSTSDISSL.ES.G-----------.DREVVRV..C...Q.ERRVS	DQ206327	n/a	0.51	
		**POU4 family**		0.99	1.00	
POU4F1	Hs	K..K.TSIAAPEKRS..AY.AV----QPRPSSEKIAA..EK.D-----------.KKNVVRV..C.Q.Q.Q.RMK	NP 006228			
POU4F2	Hs	K..K.TSIAAPEKRS..AY.AI----QPRPSSEKIAA..EK.D-----------.KKNVVRV..C.Q.Q.Q.RMK	NP 004566			
POU4F3	Hs	...K.TSIAAPEKRS..AY.AI----QPRPSSEKIAA..EK.D-----------.KKNVVRV..C.Q.Q.Q.RMK	NP 002691			
acj6	Dm	K..K.TSIAAPEKRS..AY.AV----QPRPSGEKIAA..EK.D-----------.KKNVVRV..C.Q.Q.Q.RIV	NP 727845			7;175
**POU4 (124)**	Nv	K..K.TSIGAAEKRS..AY.AM----.PRPSSDKIAS..EK.D-----------.SKNVVRV..C.Q.Q.K.RMK	DQ206292			
		**POU5 family** (found in human only)		0.84	0.95	
POU5F1	Hs	...K.TSIENRVRGN..NL.LQ----CPKP.LQQISH..QQ.G-----------.EKDVVRV..C...Q.G.RSS	NP 002692			130
FLJ25680	Hs	G.WR.ASRE.RIGNS...F.QR----CPKP.PQQISH..GC.Q-----------.QKDVVRV..Y..S.MGSRPT	NP 694948			
		**POU6 family**		0.6	0.30	
POU6F1	Hs	KRKR.TSF.PQAIEA.NAY.EK----.PLP.GQEIT...KE.N-----------YDREVVRV..C...QTL.NTS	NP 002693			
POU6F2	Hs	KRKR.TSF.PQALEI.NAH.EK----.THPSGQEMT...EK.N-----------YDREVVRV..C.K.QAL.NTI	NP 009183			
CG11641	Dm	KRKR.TSF.PQALEL.NAH.ER----.THPSGTEITGL..Q.G-----------YEREV.R...C.K.QAL.NTV	NP 610377			
**NVHD084**	Nv	KRKR.TSFSNEA.RL.ISH.EQ----.PKPSSSEIAQ..SK.G-----------.EPVTVRV..C..KQML.RMA	DQ206280			

PRD CLASS

Gene	Sp	10 20 30 40 50 60 70<br>RKRGRQTYTRYQTLELEKEFHF----NRYLTRRRRIEIAHALC-----------LTERQIKIWFQNRRMKWKKEN	Accession	NJ BP	Bayes LnL	Intron Pos
		**ALX 3/4 (CART1) family** (not monophyletic on Bayes tree)		0.85	n/a	
CART1	Hs	KR.H.T.F.SL.LE....V.QK----TH.PDVYV.EQL.LRTE-----------...ARVQV......A..R.RE	NP 008913			139
ALX3	Hs	KR.N.T.FSTF.LE....V.QK----TH.PDVYA.EQL.LRTD-----------...ARVQV......A..R.RE	NP 006483			136
ALX4	Hs	KR.N.T.F.S..LE....V.QK----TH.PDVYA.EQL.MRTD-----------...ARVQV......A..R.RE	NP 068745			139
**CART1 (120)**	Nv	KR.N.T.F.A..LE.M.RV.QK----TH.PDVYT.EQL.LRCA-----------...ARVQV......A..R.RE	DQ206256			139
		**Anf family**		n/a	n/a	
HESX1	Hs	GR.P.TAF.QN.IEV..NV.RV----.C.PGIDI.EDL.QK.N-----------.E.DR.Q.......A.L.RSH	NP 003856			37:139
		**Aristaless (AL) family** (not monophyletic on Bayes tree)		0.16	n/a	
ARX	Hs	QR.Y.T.F.S..LE...RA.QK----TH.PDVFT.E.L.MR.D-----------...ARVQV......A..R.RE	NP 620689			91:136
al	Dm	QR.Y.T.F.SF.LE....A.SR----TH.PDVFT.E.L.MKIG-----------...AR.QV......A..R.QE	NP 722629			91:136
Pph13	Dm	QR.Y.T.FNTL.LQ...RA.QR----TH.PDVFF.E.L.VRID-----------...ARVQV......A..R.QE	NP 477330			
**NVHD100**	Nv	QR.Y.T.F.S..LE...RA.AK----TH.PDVFT.EAL.VKID-----------...ARVQV......A..R.RE	DQ206234			139
		**ARIX family**		0.76	0.99	
ARIX	Hs	QR.I.T.F.SA.LK...RV.AE----TH.PDIYT.E.L.LKID-----------...ARVQV......A.FR.QE	NP 005160			136
PHOX2B	Hs	QR.I.T.F.SA.LK...RV.AE----TH.PDIYT.E.L.LKID-----------...ARVQV......A.FR.QE	NP 003915			136
PHDP	Dm	QR.I.T.F.SN.LN....I.LE----TH.PDIYT.E...SK.H-----------...ARVQV......A.FR.QE	NP 523834			142
		**CEH10 family**		0.92	0.65	
CHX10	Hs	KR.H.TVF.AH.LE....A.SE----AH.PDVYA.EML.VKTE-----------.P.DR.QV......A..R.RE	NP 878314			136
VSX1	Hs	KR.H.TVF.AH.LE....A.SE----AH.PDVYA.EML.VKTE-----------.P.DR.QV......A..R.RE	NP 055403			136
CG4136	Dm	.RH..TIF.SS.LE....A.KE----AH.PDVSA.ELLSMKTG-----------.A.DR.QV.Y....A..R.TE	NP 572232			136
CG15782	Dm	.RHS.TIF.S..LEK..EA.KE----AH.PDVYA.EMLSLKTE-----------.P.DR.QV......A..R.TE	NP 572230			
**CEH10 (085)**	Nv	KRRH.TIF.N..LE....A.KE----SH.PDVYA.ENLSLKID-----------.P.DR.QV......A..R.KE	DQ206232			139

PRD CLASS (continued)

Gene	Sp	Sequence (10 20 30 40 50 60 70)	Accession	NJ BP	Bayes LnL	Intron Pos
		RKRGRQTYTRYQTLELEKEFHF----NRYLTRRRRIEIAHALC-----------LTERQIKIWFQNRRMKWKKEN				
		**DMBX family**		**0.33**	**0.82**	
DMBX1	Hs	QR.S.TAF.AQ.LEA...T.QK----TH.PDVVM.ERL.MCTN-----------.P.ARVQV..K...A.FR.KQ	NP_671725			136
**DMBXa (090)**	Nv	IR.T.T.FNQF.LDT..RA.SR----TH.PDVLL.EQL.VYTN-----------.P.SR.QV..K...A.YR.SK	DQ206233			139
**DMBXb (063)**	Nv	SR.T.TAF.HQ.LTA...V.SK----TH.PDVEV.EQL.TSTN-----------.Q.AR.QV..K...A.YR.DQ	DQ206249			139
**DMBXc (086)**	Nv	PR.M.TCF.P..LQV..NT.CN----TH.PDVML.EQL.SYVN-----------.P.AR.QV..K...A.HR.ND	DQ206253			139
**DMBXe (064)**	Nv	HR.T.TAF.HQ.LQI..ST.SK----TH.PDVVM.EQL.AYIN-----------IP.SR.QV..K...A.YR.QV	DQ206250			139
**DMBXf (003)**	Nv	HR.T.TAF.HQ.LQI..ST.SK----TH.PDVVM.EQL.AYIN-----------IP.SR.QV..K...A.YR.QV	DQ206238			139
**DMBXd (151)**	Nv	SQIT.TRF.P...QV.NET.SS----SA.IDASTCGQL.RL.G-----------ISS.S.QV..K.K.Y.LRIQA	DQ301956	**0.02**		139
		**DUX (SIAMOIS) family**		**0.21**	**0.35**	
DUX1	Hs	GR.K.TAI.GS..AL.LRA.EK----D.FPGIAA.E.L.RETG-----------.P.SR.Q.......ARHRGQS	NP_036278			
DUX2	Hs	GQ.R.LVW.PS.SKA.QAC.ER----.P.PGITT.ERL.Q.IG-----------IP.PRVQ.L...ERSCQLRQH	NP_036279			
DUX3a	Hs	GR.MILLS.PS.SDA.RAC.ER----.L.PGIATKEQL.QGID-----------IP.PRVQ.....E.SCQLRQH	NP_036280			
DUX3b	Hs	GR.K.TAI.GS..AL.LRA.EK----D.FPGIPA.E.L.RETG-----------.P.SR.QL......ARHWGQS	NP_036280			
DUX4a	Hs	GRPR.LVW.PS.SEA.RAC.ER----.P.PGIAT.ERL.Q.IG-----------IP.PRVQ.....E.SRQLRQH	NP_149418			
DUX4b	Hs	GR.K.TAV.GS..AL.LRA.EK----D.FPGIAA.E.L.RETG-----------.P.SR.Q.......ARHPGQG	NP_149418			
DUX5	Hs	GR.K.TAI.GS..AL.LRA.EK----D.FPGIAA.E.L.RETG-----------.P.SR.Q.......ARHRGQS	NP_036281			
Loc401860a	Hs	FW.N.IQ.NQS.KDI.QSW.QH----DPFPDKAA.EQL.KEIG-----------VP.SN.QV..K.Y.V.QR.LD	XP_377445			139
Loc401860b	Hs	ARQKQTFI.WT.KNR.VQA.ER----.PFPDIAT.KK..EQTG-----------.Q.SR.QMW..KQ.SLYL.KS	XP_377445			139
Loc399839a	Hs	GR.R.LVW.PS.SEA.RAC.ER----.P.PGIAT.ERL.Q.IG-----------IP.PRVQ.....E.SRQLRQH	XP_374852			
Loc399839b	Hs	GR.K.TAV.GS..AL.LRA.EK----D.FPGIAA.E.L.RETG-----------.P.SR.Q.......ARHPGQG	XP_374852			
Loc440014	Hs	GR.K.TAV.GF..AL.LRA.EK----D.FPGIAA.E.L.RETG-----------.P.SR.Q.......ARHPGQG	XP_495855			
Loc440015a	Hs	GR.R.LVW.PS.SEA.RAC.ER----.P.PGIAT.ERL.Q.IG-----------IP.PRVQ.....E.SRQLRQH	XP_495856			
Loc440015b	Hs	GR.K.TAV.GS..AL.LRA.EK----D.FPGIAA.E.L.RETG-----------.P.SR.Q.......ARHPGQG	XP_495856			
Loc440016a	Hs	GR.R.LVW.PS.SEA.RAC.ER----.P.PGIAT.ERL.Q.IG-----------IP.PRVQ.....E.SRQLRQH	XP_495857			
Loc440016b	Hs	GR.K.TAV.GS..AL.LRA.EK----D.FPGIAA.E.L.RETG-----------.P.SR.Q.......ARHPGQG	XP_495857			
Loc440017a	Hs	GR.R.LVW.PS.SEA.RAC.ER----.P.PGIAT.ERL.Q.IG-----------IP.PRVQ.....E.SRQLRQH	XP_495858			
Loc440017b	Hs	GR.K.TAV.GS..AL.LRA.EK----D.FPGIAA.E.L.RETG-----------.P.SR.Q.......ARHPGQG	XP_495858			
Loc441056a	Hs	GR.R.LVW.PS.SEA.RAC.ER----.P.PGIAT.ERL.Q.IG-----------IP.PRVQ.....E.SRQLRQH	XP_496731			
Loc441056b	Hs	GR.K.TAV.GS..AL.LRA.EK----D.FPGIAA.E...RETG-----------.P.SRIQ.......ARHPGQG	XP_496731			
**DUXa (038)**	Nv	HR.Q.TFFSKE..VI..GA.QY----E.FPGIQI.EKL.RE.D-----------ID.SR.QV......SRQNRQK	DQ206244			139
**DUXb (005)**	Nv	HR.Q.TFFSKE..VI..GA.QY----E.FPGIQI.EKL.RE.D-----------ID.SR.QV......SRQNRQK	DQ206239			139
**DUXc (011)**	Nv	HR.Q.TFFSKE..VI..GA.QY----E.FPGIQI.EKL.RE.D-----------ID.SR.QV......SRQNRQK	DQ206241			139
		**GSC family**		**0.71**	**0.47**	
GSC	Hs	KR.H.TIF.DE.LEA..NL.QE----TK.PDVGT.EQL.RKVH-----------.R.EKVEV..K...A..RRQK	NP_776248			139
GSCL	Hs	TR.H.TIFSEE.LQA..AL.VQ----.Q.PDVST.ERL.GRIR-----------.R.ERVEV..K...A..RHQK	NP_005306			139
Gsc	Dm	KR.H.TIF.EE.LEQ..AT.DK----TH.PDVVL.EQL.LKVD-----------.K.ERVEV..K...A..R.QK	NP_476949			142
**GSC (068)**	Nv	KR.H.TIF.E-EQ....TT.QK----TH.PDVLL.E.L.MKVD-----------.K.ERVEV..K...A..R.QK	DQ206230			139
		**Homeobrain family**		**0.99**	**0.99**	
hbn	Dm	VR.S.T.F.TF.LHQ..RA.EK----TQ.PDVFT.EDL.MR.D-----------.S.ARVQV......A..R.RE	NP_788420			7;136
**HBN (079)**	Nv	VR.S.T.F.T..LHQ..RA.EK----TQ.PDVFT.E.L.LR.D-----------.S.ARVQV......A..R.RE	DQ206231			139
		**MIX family** (appears unique to vertebrates)		**n/a**	**n/a**	
MIXL1	Hs	QR.K.TSFSAE.LQL..LV.RR----T..PDIHL.ERL.ALTL-----------.P.SR.QV......A.SRRQS	NP_114150			136
		**OTP family**		**1.00**	**0.94**	
OTP	Hs	Q..H.TRF.PA.LN...RS.AK----TH.PDIFM.E.L.LRIG-----------...SRVQV......A....RK	NP_115485			136
otp	Dm	Q..H.TRF.PA.LN...RC.SK----TH.PDIFM.E...MRIG-----------...SRVQV......A....RK	NP_995909			136
**OTP (047)**	Nv	Q..H.TRF.PA.LN...RC.AR----TH.PDVFM.E.L.ARIG-----------...SRVQV......A....RK	DQ206247			139

PRD CLASS (continued)

Gene	Sp	Sequence (10 20 30 40 50 60 70)	Accession	NJ BP	Bayes LnL	Intron Pos
		RKRGRQTYTRYQTLELEKEFHF----NRYLTRRRRIEIAHALC-----------LTERQIKIWFQNRRMKWKKEN				
		**OTX family**		0.97	1.00	
OTX1	Hs	QR.E.T.F..S.LDV..AL.AK----T..PDIFM.E.V.LKIN-----------.P.SRVQV..K...A.CRQQQ	NP 055377			136
OTX2	Hs	QR.E.T.F..A.LDV..AL.AK----T..PDIFM.E.V.LKIN-----------.P.SRVQV..K...A.CRQQQ	NP 068374			139
CRX	Hs	QR.E.T.F..S.LE...AL.AK----TQ.PDVYA.E.V.LKIN-----------.P.SRVQV..K...A.CRQQR	NP 000545			142
oc	Dm	QR.E.T.F..A.LDV..AL.GK----T..PDIFM.E.V.LKIN-----------.P.SRVQV..K...A.CRQQL	NP 511091			139
**OTXa (114)**	Nv	QR.E.T.F.KN.LEV..EL.AK----T..PDIFM.E.V.IKIN-----------.P.SRVQV..K...A.TRQLE	DQ206236			139
**OTXb (123)**	Nv	QR.E.T.F.KN.LEI..EL.AK----T..PDIFM.E.V.IKIN-----------.P.SRVQV..K...A.ARQQA	DQ206257			139
**OTXc (078)**	Nv	QR.E.T.F.KN.LEI..EL.AK----T..PDIFM.E.V.IKIN-----------.P.SRVQV..K...A.LRQLS	DQ206252			139
		**PAX3/7 family**		0.56	0.59	
PAX3	Hs	QR.S.T.F.AE.LE...RA.ER----TH.PDIYT.E.L.QRAK-----------...ARVQV..S...AR.R.QA	NP 852123			139
PAX7	Hs	QR.S.T.F.AE.LE....A.ER----TH.PDIYT.E.L.QRTK-----------...ARVQV..S...AR.R.QA	NP 002575			136
gsb	Dm	QR.S.T.FSND.IDA..RI.AR----TQ.PDVYT.E.L.QSTG-----------...ARVQV..S...ARLR.QL	NP 523863			
gsb-n	Dm	QR.S.T.F.AE.LEA..RA.SR----TQ.PDVYT.E.L.QTTA-----------...AR.QV..S...ARLR.HS	NP 523862			
prd	Dm	QR.C.T.FSAS.LD...RA.ER----TQ.PDIYT.E.L.QRTN-----------...AR.QV..S...ARLR.QH	NP 723721			
**NVHD014**	Nv	QR.S.TKF.SK.VD....A.LK----TQ.PDVYT.E.L.QR.N-----------...ARVQV..S...ARLR.KK	DQ206242			139
**NVHD101**	Nv	PR.S.TRF.VS..D...RA.RK----TH.PDIYA.E.L.QR.G-----------.S.ARVQV..S...ARLR.ER	DQ206226			139
		**PAX 4/6 family**		0.01	0.25	
PAX4	Hs	GH.N.TIFSPS.AEA.....QR----GQ.PDSVA.GKL.T.TS-----------.P.DTVRV..S...A..RRQE	NP 006184			55;142
PAX6	Hs	LQ.N.TSF.QE.IEA.....ER----TH.PDVFA.ERL.AKID-----------.P.AR.QV..S...A..RR.E	NP 001595			55;136
CXorf43	Hs	ALQD.TQFSDRDLAT.K.YWDNG---MTS.GSVC.EK.EAVATELN--------VDCEIVRT.IG...R.Y.LNV	NP 653258			91
eyg	Dm	FR.N.T.FSPE.LE......DK----SH.PCVST.ERLSSRTS-----------.S.ARVQV..S...A..RRH	NP 524042			135
toe	Dm	FR.N.T.FSPE.LD......DK----SH.PCVNT.EKL.ARTA-----------.S.ARVQV..S...A..RRHQ	NP 524041			136
toy	Dm	LQ.N.TSFSNE.IDS.....ER----TH.PDVFA.ERL.DKIG-----------.P.AR.QV..S...A..RR.E	NP 524638			142
ey	Dm	LQ.N.TSF.ND.IDS.....ER----TH.PDVFA.ERL.GKIG-----------.P.AR.QV..S...A..RR.E	NP 726607			55
**PAX4/6 (022)**	Nv	LR.N.T.F.PD.LEM.....EK----SH.PDVAT.E.L.NKID-----------MS.ARVQV..S...A..RRHQ	DQ206243			139
**NVHD148**	Nv	SR.Q.TNF.DE.IEK...V.EK----TH.PDVFT.E.L.QQVN-----------.S.AR.QV.YS...A..R.EG	DQ206260	0.01	n/a	139
		**PRX family**		0.87	0.76	
PMX1	Hs	QR.N.T.FNSS.LQA..RV.ER----TH.PDAFV.EDL.RRVN-----------...ARVQV......A.FRRNE	NP 008833			136
PMX2	Hs	QR.N.T.FNSS.LQA..RV.ER----TH.PDAFV.E.L.RRVN-----------.S.ARVQV......A.FRRNE	NP 057391			136
CG9876	Dm	PR.N.T.FSSA.LTA...V.ER----TH.PDAFV.E.L.TKVH-----------.S.ARVQV......A.FRRNE	NP 611756			136
		**PTX family**		0.99	1.00	
PITX1	Hs	QR.Q.THF.SQ.LQ...AT.QR----...PDMSM.E...VWTN-----------...PRVRV..K...A..R.RE	NP 002644			139
PITX2	Hs	QR.Q.THF.SQ.LQQ..AT.QR----...PDMST.E...VWTN-----------...ARVRV..K...A..R.RE	NP 000316			139
PITX3	Hs	QR.Q.THF.SQ.LQ...AT.QR----...PDMST.E...VWTN-----------...ARVRV..K...A..R.RE	NP 005020			139
Ptx1	Dm	QR.Q.THF.SQ.LQ...HT.SR----...PDMST.E...MWTN-----------...ARVRV..K...A..R.RE	NP 996314			142
**PTX (105)**	Nv	QR.Q.THF.SF.LQQ..GT.GR----...PDMQM.E...LYTN-----------...ARVRV..K...A..R.KE	DQ206235			139
		**REPO family**		0.6	0.73	
repo	Dm	K.KT.T.F.A..LE...RA.ER----AP.PDVFA.E.L.IK.N-----------.S.SRVQV......A..R.HE	NP 477026			13
**REPO (118)**	Nv	KT.Y.T.FSQ..IE...RA.DK----AP.PDVFA.E.L.AK.G-----------...AR.QV......A..R.RE	DQ206237			139
		**RX family**		0.96	1.00	
RX	Hs	HR.N.T.F.T..LH...RA.EK----SH.PDVYS.E.L.GKVN-----------.P.VRVQV......A..RRQE	NP 038463			136
MGC15631	Hs	HR.N.T.F.T..LHQ..RA.EA----SH.PDVYS.E.L.AKVH-----------.P.VRVQV......A..RRQE	NP 116142			136
Rx	Dm	HR.N.T.F.T..LH...RA.EK----SH.PDVYS.E.L.MKVN-----------.P.VRVQV......A..RRQE	NP 726006			136
**RX (129)**	Nv	LR.N.T.F.TF.LH...RA.EK----SH.PDVYT.E.L.LKIS-----------.P.VRVQV......A..RRQE	DQ206258			139
		**SHOX (OG12) family**		0.99	0.99	
SHOX	Hs	QR.S.TNF.LEQLN...RL.DE----TH.PDAFM.E.LSQR.G-----------.S.ARVQV......AKCRKQE	NP 000442			139
SHOX20.61	Hs	QR.S.TNF.LE.LN...RL.DE----TH.PDAFM.E.LSQR.G-----------.S.ARVQV......A.CR.QE	NP 003021			139

PRD CLASS (continued)

Gene	Sp	10 20 30 40 50 60 70<br>RKRGRQTYTRYQTLELEKEFHF----NRYLTRRRRIEIAHALC-----------LTERQIKIWFQNRRMKWKKEN	Accession	NJ BP	Bayes LnL	Intron Pos
		**UNC4 family** (may be present as a pseudogene in human)		0.59	1.00	
unc-4	Dm	.R.S.TNFNSW.LE...RA.SA----SH.PDIFM.EAL.MR.D-----------.K.SRVAV......A.VR.RE	NP 573242			142
OdsH	Dm	KR...TNFNSW.LR...RV.QG----SH.PDIFM.EAL.TK.D-----------.M.GR.AV......A..R.QE	NP 523389			139
**UNC4 (059)**	Nv	.M.V.TNFSPW.LE...HA.ET----TH.PDVFM.EAL.MR.D-----------...ARVQV......A..R.RE	DQ206229			139
		**PRD CLASS / Family membership ambiguous**		n/a	n/a	
Loc91464	Hs	KR.V.T.F.TE.LH....I...----TH.PDVHI.SQL.ARIN-----------.P.ARVQ.....Q.A..R.QE	NP_001008494			136
PROP1	Hs	.R.H.T.FSPV.LEQ..SA.GR----.Q.PDIWA.ESL.RDTG-----------.S.AR.QV......A.QR.QE	NP_006252			136
CG11294	Dm	QR.N.T.F.PQ.LQ...AL.QK----TH.PDVFL.E.V.LRIS-----------.S.ARVQV......A..R.QA	NP_572500			136
CG32532	Dm	.R.H.T.F.QE.LA...AA.AK----SH.PDIYC.E.L.RTTK-----------.N.AR.QV......A.YR.QE	NP_608318			28
**NVHD002**	Nv	QR.N.T.F.KQ.LQ....V.EK----KH.PDIAL.E.L.AKIN-----------IS.AR.QV?????????????	DQ206222			139
**NVHD031**	Nv	.R.C.TVFSTE.LAI..EG.QK----QHFPDNKL.QA..TRAG-----------.P.DRVQV......A.E.RLL	DQ206265			
**NVHD037**	Nv	KR.H.T.FSKL.LDS..EA.SR----SQ.PDVFT.EQL.KRIN-----------.N.ARVQV......A.HR.QE	DQ206228			139
**NVHD052**	Nv	I..K.RNL.KR.QKI..TAYST----IK.P.IED.QRLETSTQ-----------.S.DR.QV......AKDRRLQ	DQ206271			
**NVHD058**	Nv	HR.K.T.F.HD.LQLM.AY..N----...PGIEQ.EGL.EKIQ-----------VS.SRLQV......S..R.HQ	DQ206248			139
**NVHD074**	Nv	QR.N.TKF.A..LEQ..DAYQK----AK.PDVQA.ETL.QR.G-----------VA.SRVQV..S...S.G.RKE	DQ206251			139
**NVHD146**	Nv	G..Y.A.FDKA.IFQM.RV.LL----.H.PDVAA.S.LSRRTG-----------.S.S.VQ.......A..R.QQ	DQ206259			139

PROS CLASS (found in *Drosophila* but not included in present study)

Gene	Sp	10 20 30 40 50 60 70<br>RKRGRQTYTRYQTLELEKEFHF----NRYLTRRRRIEIAHALC-----------LTERQIKIWFQNRRMKWKKEN	Accession	NJ BP	Bayes LnL	Intron Pos
Prox1	Hs	GSAMQEGLSPNHLKKAKLM.FY----T..PSSNMLKTYFSDVKFNR--------CITS.LIK..S.FREFYYIQM	NP_002754			118

SINE CLASS

Gene	Sp	10 20 30 40 50 60 70<br>RKRGRQTYTRYQTLELEKEFHF----NRYLTRRRRIEIAHALC-----------LTERQIKIWFQNRRMKWKKEN	Accession	NJ BP	Bayes LnL	Intron Pos
		**SIX 1/2 family**		0.91	0.78	
SIX1	Hs	GEETSYCFKEKSRGV.REWYAH----.P.PSP.EKR.L.E.TG-----------..TT.VSN..K...QRDRAAE	NP 005973			
SIX2	Hs	GEETSYCFKEKSRSV.REWYAH----.P.PSP.EKR.LTE.TG-----------..TT.VSN..K...QRDRAAE	NP 058628			
so	Dm	GEETSYCFKEKSRSV.RDWYSH----.P.PSP.EKRDL.E.TG-----------..TT.VSN..K...QRDRAAE	NP 476733			130
**SIX1/2 (073)**	Nv	GEETSYCFKEKSRNI.REWYSH----.P.PSP.EKR.L.EGTG-----------..TT.VSN..K...QRDRAAE	DQ206326			
		**SIX 3/6 family**		0.99	1.00	
SIX3	Hs	GEQKTHCFKERTRSL.REWYLQ----DP.PNPSKKR.L.Q.TG-----------..PT.VGN..K...QRDRAAA	NP 005404			
SIX6	Hs	GEQKTHCFKERTRNL.REWYLQ----DP.PNPSKKR.L.Q.TG-----------..PT.VGN..K...QRDRAAA	NP 031400			
Optix	Dm	GEQKTHCFKERTRSL.REWYLQ----DP.PNPTKKR.L.K.TG-----------.NPT.VGN..K...QRDRAAA	NP 524695			
**SIX3/6 (128)**	Nv	GEQKTHCFKERTRSL.REWYLQ----DP.PNPTKKR.L.Q.TG-----------..PT.VGN..K...QRDRAAA	DQ206294			
		**SIX 4/5 family**		0.55	0.95	
SIX4	Hs	GEETVYCFKEKSRNA.KELYKQ----...PSPAEKRHL.KITG-----------.SLT.VSN..K...QRDRNPS	NP 059116			
SIX5	Hs	GEETVYCFKERSRAA.KACYRG----...P.PDEKRRL.TLTG-----------.SLT.VSN..K...QRDRTGA	NP 787071			
Six4	Dm	GEETVYCFKEKSRNA.KDCYLT----...P.PDEKKTL.KKTG-----------..LT.VSN..K...QRDRTPQ	NP 649256			
**SIX4/5a (030)**	Nv	GEETVYCFKEKARAA.KDCYEQ----.K.P.PQEKRL..KQTN-----------..LK.VSN..K...QRGRIPS	DQ206264			
**SIX4/5b (061)**	Nv	GEKTIYFFKEKVRTV.KECYEH----KK.P.LKEKRV..TQTN-----------..L..VRN..R...HRDRISS	DQ206322	n/a	0.95	
		**SINE CLASS / family membership ambiguous**				
**NVHD093**	Nv	GEETTYCFKEKSRSI.N.AYVD----SP.P.P.EKH.L.KMTD-----------..VT.VSN..K.K.QRVRAAE	DQ206281			

TALE CLASS (not monophyletic on neighbor-joining tree)

	Sp.	10 20 30 40 50 60 70<br>RKRGRQTYTRYQTLELEKEFHF----NRYLTRRRRIEIAHALC-----------LTERQIKIWFQNRRMKWKKEN	Accession	NJ BP	Bayes LnL	Intron Pos
**IRX family**				0.54	0.94	
C10orf48	Hs	KVRHKRQALQDMARP.KQWLYKHRD-.P.P.KTEK.LL.LGSQ-----------M.LV.VSN..A.A.RRL.NTV	NP_775847			142
IRX1	Hs	DPGRPKNA.RES.ST.KAWLNEHRK-.P.P.KGEK.ML.IITK-----------M.LT.VST..A.A.RRL....	NP_077313			
IRX2	Hs	DPAY.KNA..DA.AT.KAWLNEHRK-.P.P.KGEK.ML.IITK-----------M.LT.VST..A.A.RRL....	NP_150366			
IRX3	Hs	DPSRPKNA.RES.ST.KAWLNEHRK-.P.P.KGEK.ML.IITK-----------M.LT.VST..A.A.RRL....	NP_077312			
IRX4	Hs	SGTR.KNA..ET.ST.KAWLQEHRK-.P.P.KGEK.ML.IITK-----------M.LT.VST..A.A.RRL....	NP_057442			
IRX5	Hs	DPAY.KNA.RDA.AT.KAWLNEHRK-...P.KGEK.MLAIITK-----------M.LT.VST..ANA.RRL....	NP_005844			
IRX6	Hs	GAGR.KNA..ET.ST.KAWLNEHRK-.P.P.KGEK.ML.IITK-----------M.LT.VST..A.A.RRL....	NP_077311			
ara	Dm	LAAR.KNA..ES.AT.KAWLNEHKK-.P.P.KGEK.ML.IITK-----------M.LT.VST..A.A.RRL....	NP_524045			
caup	Dm	LAAR.KNA..ES.AT.KAWLSEHKK-.P.P.KGEK.ML.IITK-----------M.LT.VST..A.A.RRL....	NP_524046			
mirr	Dm	NGAR.KNA..ET.ST.KAWLNEHKK-.P.P.KGEK.ML.IITK-----------M.LT.VST..A.A.RRL....	NP_524047			
**IRX (108)**	Nv	AGAR.KNA..ET.ST.KAWLFEHRK-.P.P.KGEK.ML.ILTK-----------M.LT.VST..A.A.RRL....	DQ206288			
**MEIS family**				0.76	1.00	
MEIS1	Hs	.HKK.GIFPKVA.NIMRAWLFQHLT-HP.PSEEQKKQL.QDTG------------..IL.VNN..I.A.RRIVQPM	NP_002389			76:154
MEIS2	Hs	.QKK.GIFPKVA.NIMRAWLFQHLT-HP.PSEEQKKQL.QDTG------------..IL.VNN..I.A.RRIVQPM	NP_002390			76:154
MEIS3	Hs	.NKK.GIFPKVA.NIMRAWLFQHLS-HP.PSEEQKKQL.QDTG------------..IL.VNN..I.A.RRIVQPM	NP_001009813			
hth	Dm	NQKK.GIFPKVA.NI.RAWLFQHLT-HP.PSEDQKKQL.QDTG------------..IL.VNN..I.A.RRIVQPM	NP_476577			76:154
**MEIS (107)**	Nv	SQKK.GIFPKAA.NIMKAWLFQHLT-HP.PSEEQKRSL.QETG------------..IL.VNN..I.A.RRIVQPM	DQ206227			67:144
**PBX family**				0.96	1.00	
PBX1	Hs	AR.K.RNFNKQATEI.NEY.YSHLS-.P.PSEEAKE.L.KKCG-----------I.VS.VSN..G.K.IRY..NI	NP_002576			142
PBX2	Hs	AR.K.RNFSKQATEV.NEY.YSHLS-.P.PSEEAKE.L.KKCG-----------I.VS.VSN..G.K.IRY..NI	NP_002577			139
PBX3	Hs	AR.K.RNFSKQATEI.NEY.YSHLS-.P.PSEEAKE.L.KKCS-----------I.VS.VSN..G.K.IRY..NI	NP_006186			142
PBX4	Hs	AR.K.RNFSKQA.EV.NEY.YSHLN-.P.PSEEAKE.L.RKGG------------..IS.VSN..G.K.IRY..NM	NP_079521			139
exd	Dm	AR.K.RNFSKQASEI.NEY.YSHLS-.P.PSEEAKE.L.RKCG-----------I.VS.VSN..G.K.IRY..NI	NP_523360			133
**PBX (040)**	Nv	YR.K.RNFSKQATEI.NEY.YSHLS-.P.PSEEAKE.L.RKCN-----------ISVA..SN..G.K.IRY..NI	DQ206245			133
**TGIF family**				0.86	0.90	
TGIF	Hs	KR.R.GNLPKESVQI.RDWLYEHRY-.A.PSEQEKALLSQQTH------------.STL.VCN..I.A.RRLLPDM	NP_003235			138
achi	Dm	LRKR.GNLPKTSVKI.KRWLYEHRY-.A.PSDAEKFTLSQEAN------------..VL.VCN..I.A.RRILP.M	NP_725182			
vis	Dm	...-.GNLPK-SSVKIL.RWLYHRY..A.PSDAEKFTLSQEAN------------..VL.VCN..I.A.RRILP.M	NP_523714			
**TGIF (149)**	Nv	P..R.GNLPKDSVNV.RLWLWEHRF-.A.PSEAEKQYLSK.AN------------.SVL.VCN..I.A.RRILPDM	DQ206298			
**TALE CLASS / Family membership ambiguous**				n/a	n/a	
PNX1	Hs	S.NK.GVLPKHA.NVMRSWLFQHIG-HP.P.EDEKKQ..AQTN------------..LL.VNN..I.A.RRILQPM	NP_004562			
**NVHD036**	Nv	S.KSKRGVLPKQATSIM.TWL.QHIMHP.P.EDEKRS..QQTN------------..IL.VNN..I.A.RRILQPM	DQ301954			38;115
**NVHD143**	Nv	ARKR.GKLPESARTR.MTWLFAHSN-RP.P.EEEKSNL.TITG------------..PI..NN..S.A.RRIL.KA	DQ301952			

ZF CLASS (not monophyletic on Bayes or NJ trees)

	Sp.	10 20 30 40 50 60 70<br>RKRGRQTYTRYQTLELEKEFHF----NRYLTRRRRIEIAHALC-----------LTERQIKIWFQNRRMKWKKEN	Accession	NJ BP	Bayes LnL	Intron Pos
**ZFH-1**				n/a	n/a	
zfh1	Dm	KV.V.TAINEE.QQQ.KQHYSL----.ARPS.DEFRM..AR.Q------------.DP.VVQV....N.SRER.MQ	NP_476850			
**ZFH-2**				0.89	0.99	
zfh2	Dm	N..L.T.ILPE.LNF.YECYQS----ESNPS.KMLE..SKKVN------------.KK.VVQV....S.A.D..SR	NP_524623			139
ATBF1c	Hs	D..L.T.I.PE.LEI.YQKYLL----DSNP..KMLDH...EVG------------.KK.VVQV....T.ARER.GQ	NP_008816			
**ZFHX2 family**				0.98	1.00	
ZFHX2	Hs	QR.Y.TQMSSL.LKIMKACYEA----Y.TP.MQECEVLGEEIG------------.PK.V.QV....A.A.E..AK	NP_207646			
ATBF1d	Hs	Q..F.TQM.NL.LKV.KSC.ND----YRTP.MLECEVLGNDIG------------.PK.VVQV....A.A.E..SK	NP_008816			
**ZFHX4 family**				0.99	0.99	
ATBF1b	Hs	KRSS.TRF.D..LRV.QDF.DA----.A.PKDDEFEQLSNL.N------------.PT.V.VV....A.Q.AR.NY	NP_008816			
ZFHX4	Hs	KRSS.TRF.D..LRV.QDF.DT----.A.PKDDEIEQLSTV.N------------.PT.V.VV....A.Q.AR.SY	NP_078997			

ZF CLASS (continued)

	Sp.	10 20 30 40 50 60 70<br>RKRGRQTYTRYQTLELEKEFHF----NRYLTRRRRIEIAHALC-----------LTERQIKIWFQNRRMKWKKEN	Accession	NJ BP	Bayes LnL	Intron Pos
		**ZHX FAMILY**		0.56	0.85	
ZHX1a	Hs	SFGI.AKK.KE.LA..KVSYLK----.QFPHDSEI.RLMKITG-----------..KGEI.K..SDT.YNQRNSK	NP_001017926			
ZHX1b	Hs	STGKICKK.PE.LHM.KSA.VR----TQWPSPEEYDKL.KESG-----------.ARTD.VS..GDT.YA..NG.	NP_001017926			
ZHX2	Hs	TPASDRKKTKE.IAH.KAS.LQ----SQFPDDAEVYRLIEVTG-----------.ARSE..K..SDHRYRCQRGI	NP_207646			
ZHX3a	Hs	ASIYKNKKSHE.LSA.KGS.CR----.QFPGQSEVEHLTKVTG-----------.ST.EVRK..SD..YHCRNLK	NP_055850			
ZHX3b	Hs	TPTKYKERAPE.LRA..SS.AQ----.PLPLDEELDRLRSETK-----------M.R.E.DS..SE..---..V.	NP_055850			172
		**ZF CLASS / Family membership ambiguous**				
ATBF1a	Hs	KRSS.TRF.D..LRV.QDF.DA----.A.PKDDEFEQLSNL.N-----------.PT.V.VV....A.Q.AR.NY	NP_008816			

CLASS AMBIGUOUS

	Sp.	10 20 30 40 50 60 70<br>RKRGRQTYTRYQTLELEKEFHF----NRYLTRRRRIEIAHALC-----------LTERQIKIWFQNRRMKWKKEN	Accession	NJ BP	Bayes LnL	Intron Pos
		**LASS**		0.65	0.28	
LASS3	Hs	FGIKETVRKVTPNTV..NF.KHS---T.QPLQTDIYGL.KKCN-----------.....VER..RS..NQERPSR	NP_849164			85
LASS5	Hs	GIEDSGP.QAQPNAI...V.ISI---TK.PDKK.LEGLSKQ.D-----------WNV.K.QC..RH..NQD.PPT	NP_671723			76
Lag1	Dm	GI.SSRPKKAANVPI...TYAK----STR.DKKKLVPLSKQTD-----------MS..E.ER.WRL..AQDKPST	NP_652526			91
ADNP	Hs	PKGHEDDSYEARKSFLT.Y.NK----QP.P...EIEKL.AS.W-----------.WKSD.ASH.S.K.K.CVRDC	NP_056154			
ESX1L	Hs	KR.R.TAF.QF.LQ...NF.DE----SQ.PDVVA.ERL.AR.N-----------...DRVQV......A...RNQ	NP_703149			91;139
OTEX	Hs	PRTR.TKF.LL.VE...SV.RH----TQ.PDVPT.R.L.EN.G-----------V..DKVRV..K.K.ARCRRHQ	NP_644811			91;139
PEPP-2	Hs	QQPNVHAF.PL.LQ...RI.QR----EQFPSEFL.RRL.RSMN-----------V..LAVQ...E...A..RRHQ	NP_115887			91;139
HOP	Hs	SAETASGP.EDQVEI..YN.NKV---DKHPDSTTLCL..AEAG-----------.S.EETQK..KQ.LA..RRSE	NP_115884			
LOC342900	Hs	YR.P.TRFLSK.LTA.RELLEK----TMHPSLATMGKL.SK.Q-----------.DLSVV....K.Q.A...RQQ	XP_297205			139
LOC401860c	Hs	KTKH.HKFSEELLQ..KEI.GE----.G.PDFTT.KTL.NKFD-----------CPVNV.NN....N.ARLPP.E	XP_377445			139
Loc401861	Hs	KTKH.HKFSEELLQ..KEI.GE----.C.PDYTT.KTL.IKFD----------------CPN....K.ARLPPAE	XP_377446			124:16
CG11617	Dm	SRATKRLF.PDIKRMLKDWLIRRRE-.P.PS.EEKKQL.AETG-----------..YT..CN..A.W.R.L.NSE	NP_608502			
**NVHD088**	Nv	AP.N.KNF.GD.LRD..RL.EQ----TH.PDAMT.EGL.KKLG-----------.S.ARVQ.......A.SRRLE	DQ206254	**n/a**	0.32	139
**NVHD007**	Nv	KG.QKS.LPKEA.RI.QSWLNDNLE-KP.PDAETKERLQQLTQ-----------.SKT.VNT..A.A.RRLNRNR	DQ206240			133
**NVHD045**	Nv	KG.QKS.LPKEA.RI.QSWLNDNLE-KP.PDAETKERLQQLTQ-----------.SKT.VNT..A.A.RRLNRNR	DQ206246			133
**NVHD144**	Nv	TAVPITFDRQAAEVPGSLAHAD----.P.PN..QKQ.LSV.TG-----------.SV..VEQ..V.Y.RRKGRTA	DQ301953			
