# Supplementary figures and images for: The cnidarian-bilaterian ancestor possessed at least 56 homeoboxes: evidence from the starlet sea anemone, Nematostella vectensis
Source: Genome Biol. 2006 Jul 24;7(7):R64. doi: 10.1186/gb-2006-7-7-r64 (PMC1779571; doi:10.1186/gb-2006-7-7-r64)

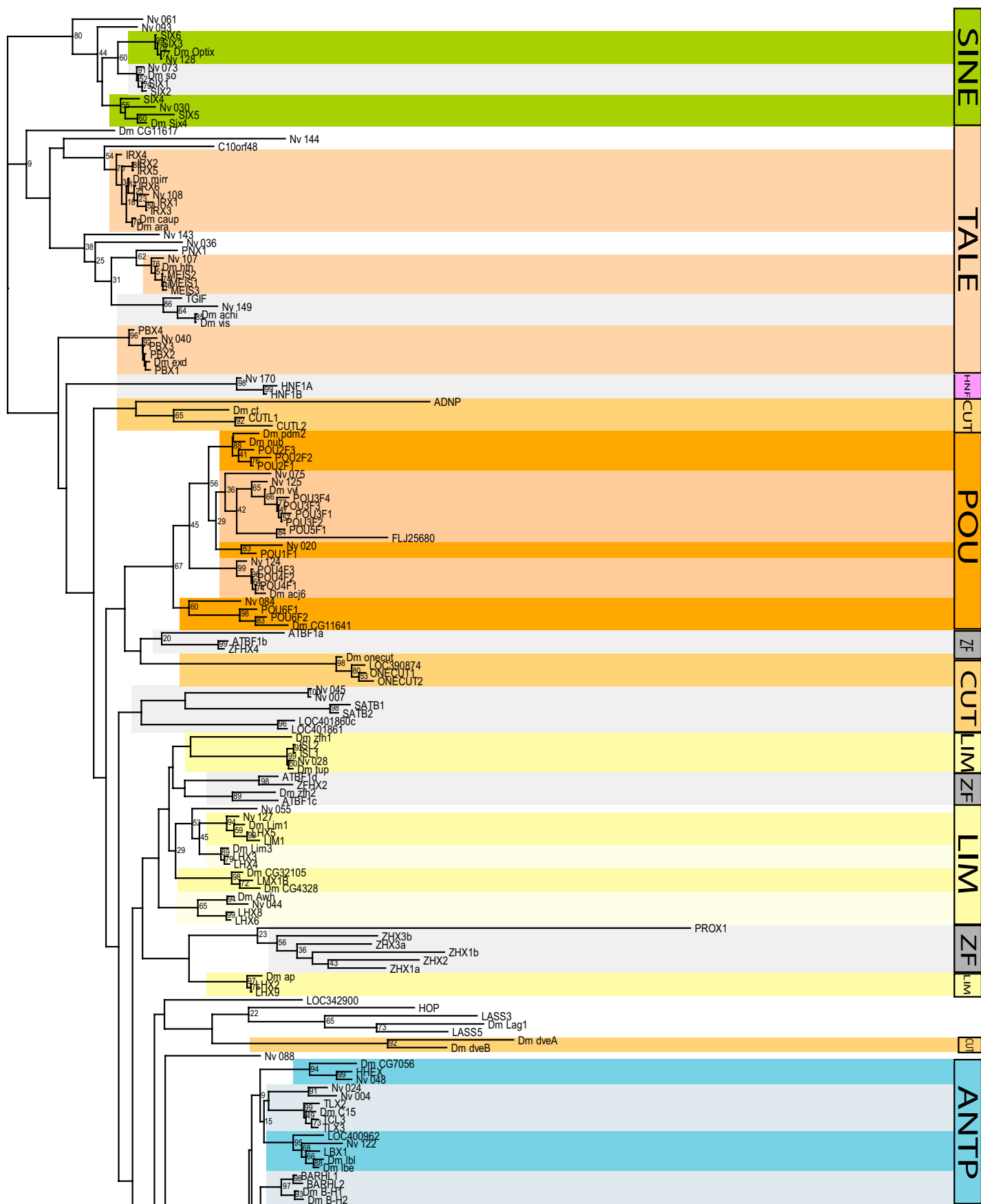

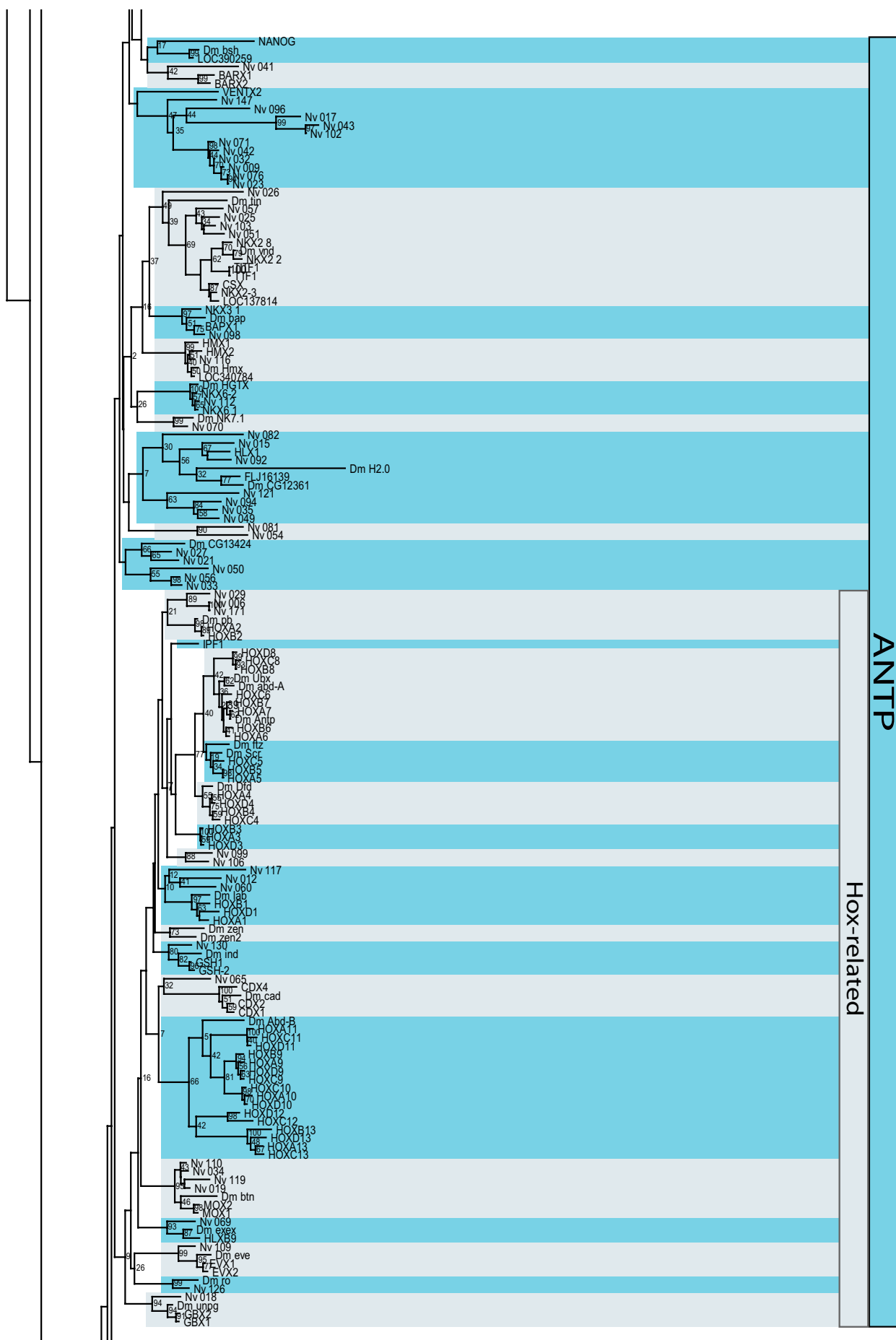

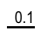

Supplement: Additional data file 2 — Neighbor-joining phylogeny depicting the relationships among 455 distinct homeodomain sequences (130 from Nematostella, 97 from Drosophila, and 228 from human). [file gb-2006-7-7-r64-S2.pdf]

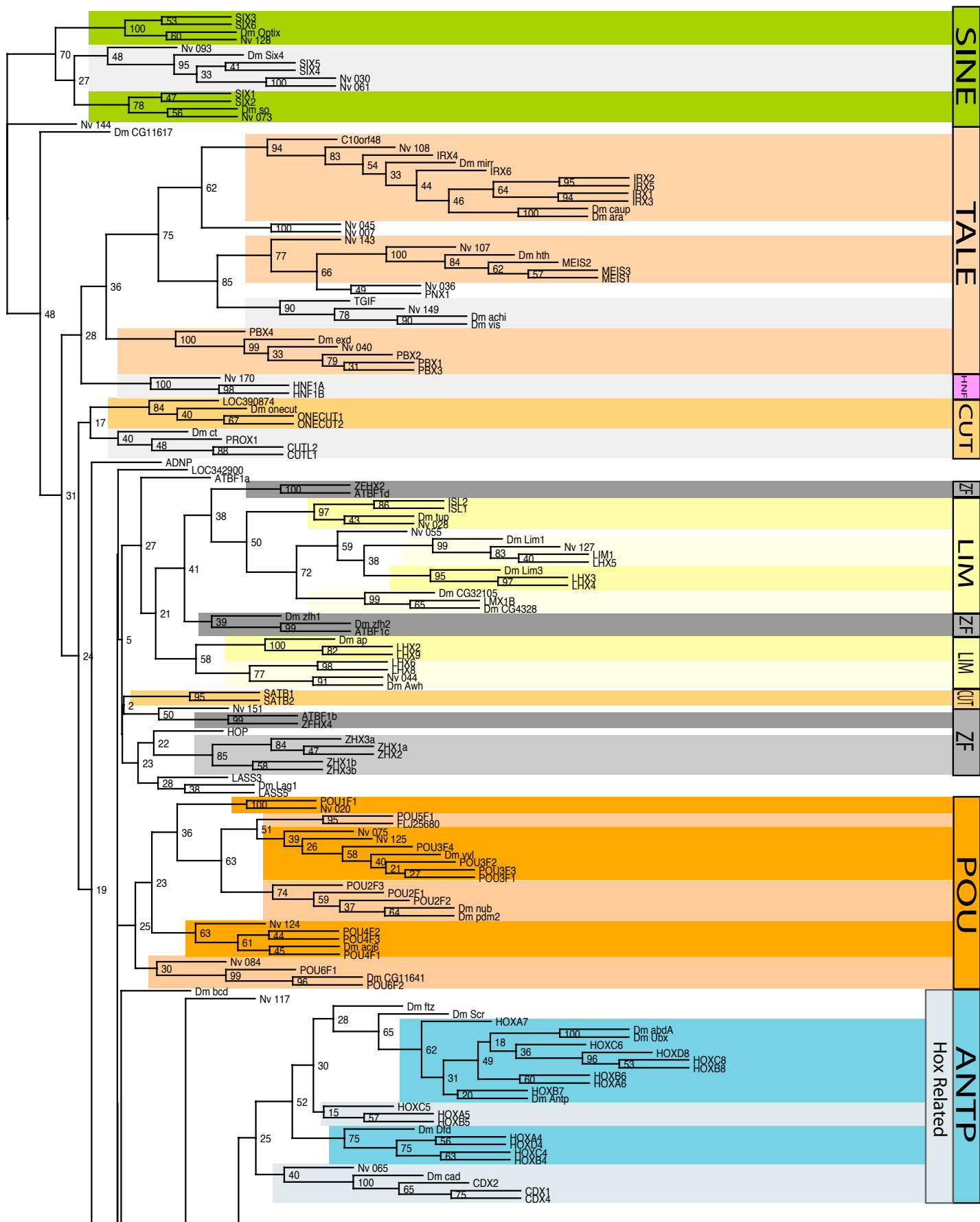

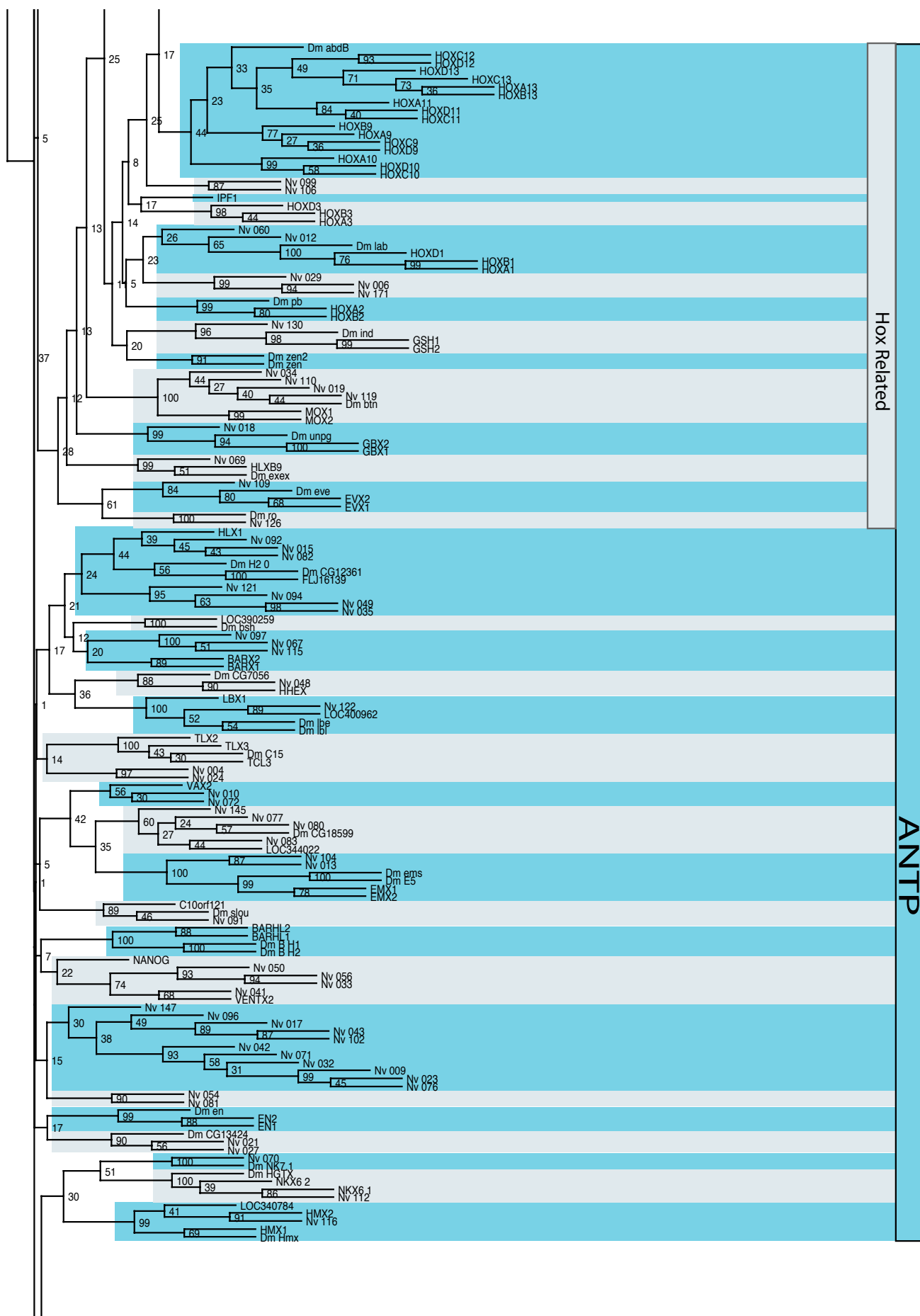

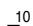

Supplement: Additional data file 3 — Bayesian phylogeny depicting the relationships among the same 455 homeodomain sequences. [file gb-2006-7-7-r64-S3.pdf]
